# NMRDSP: An Accurate Prediction of Protein Shape Strings from NMR Chemical Shifts and Sequence Data

**DOI:** 10.1371/journal.pone.0083532

**Published:** 2013-12-23

**Authors:** Wusong Mao, Peisheng Cong, Zhiheng Wang, Longjian Lu, Zhongliang Zhu, Tonghua Li

**Affiliations:** Department of Chemistry, Tongji University, Shanghai, China; University of Alberta, Canada

## Abstract

Shape string is structural sequence and is an extremely important structure representation of protein backbone conformations. Nuclear magnetic resonance chemical shifts give a strong correlation with the local protein structure, and are exploited to predict protein structures in conjunction with computational approaches. Here we demonstrate a novel approach, NMRDSP, which can accurately predict the protein shape string based on nuclear magnetic resonance chemical shifts and structural profiles obtained from sequence data. The NMRDSP uses six chemical shifts (HA, H, N, CA, CB and C) and eight elements of structure profiles as features, a non-redundant set (1,003 entries) as the training set, and a conditional random field as a classification algorithm. For an independent testing set (203 entries), we achieved an accuracy of 75.8% for S8 (the eight states accuracy) and 87.8% for S3 (the three states accuracy). This is higher than only using chemical shifts or sequence data, and confirms that the chemical shift and the structure profile are significant features for shape string prediction and their combination prominently improves the accuracy of the predictor. We have constructed the NMRDSP web server and believe it could be employed to provide a solid platform to predict other protein structures and functions. The NMRDSP web server is freely available at http://cal.tongji.edu.cn/NMRDSP/index.jsp.

## Introduction

Nuclear Magnetic Resonance (NMR) is a well-established technique that allows the determination of three-dimensional biological macromolecule structures in solution. NMR chemical shifts (CSs) give a strong correlation with local protein structures. Currently, NMR CS is exploited to predict the secondary and tertiary structures of proteins in conjunction with computational approaches. Vendruscolo et al. demonstrated it was possible to use CSs in combination with conventional molecular mechanical force field techniques to determine the conformation of proteins [1]. Shen et al. proposed a CS based structure determination protocol using an empirically optimized procedure to select protein fragments from the Protein Data Bank (PDB), in conjunction with the standard Rosetta Monte Carlo assembly and relaxation methods to generate protein structure [2]. Wishart et al. constructed a web server to rapidly generate accurate three-dimensional protein structures using only assigned NMR CSs and sequence data [3]. Raman et al. showed that structures could be accurately determined by incorporating backbone CS, residual dipolar couplings, and amide proton distances into the Rosetta protein structure modeling methodology [4]. In these studies, NMR CS was used indirectly as structural restraints to reduce the search spaces.

Essentially, NMR CS is directly related with the local structure of the protein backbone. Many studies have demonstrated that an accurate prediction of protein secondary structures could utilize NMR CSs and sequence data. Wang et al. performed two-dimension clustering analyses of NMR CS to identify protein secondary structures and the redox state of the cysteine residue [5]. Krishnan et al. presented a comprehensive overview of low-resolution structural determinants to correlate NMR CS data with protein structural data in order to provide meaningful information expeditiously [6]. Ikeda et al. presented a method for assigning ^13^C CSs and secondary structures from unresolved two-dimensional NMR spectra by spectral fitting, named reconstruction of spectra using protein local structures [7].

Besides protein secondary structure, the protein backbone dihedral angle is also one of the main research areas using NMR CS. The protein backbone dihedral angle can be expressed by angle degrees or structure alphabets. TALOS+ [8] was a widely used program used to establish an empirical relationship between ^13^C, ^15^N and ^1^H CSs with backbone torsion angles Φ and Ψ, which extended the training set of the original TALOS [9] from a database containing 20 proteins to 200 proteins. Hirst et al. independently predicted both the secondary structure and the backbone dihedral angles and combined the results in a loop to enhance each prediction reciprocally [10]. The dihedral angle space was divided into eight regions using an unsupervised clustering technique. Actually, the Ramachandran plot [11] of the protein backbone dihedral angles had been divided into distinct regions defined as shape strings [12]. Shape strings are expressed by eight characters and are considered as structural alphabets. There are several expressions of the structural alphabets that have been utilized in protein structural studies. Offmann et al. mined 16 short structural motifs to represent most of the local structural features of a protein backbone, and developed a protein structural comparison method [13]. Koehl et al. used an alphabet of 20 letters, corresponding to four residues, to find structural similarities between proteins [14]. Tuffèry et al. considered the structural alphabets as a generalization of the concept of secondary structure and recognized protein folding with an optimum alphabet size of 27 structures [15]. These coarse representations of protein structures can be used for structure comparison and sensible alignment.

In our previous studies, the predicted shape string was explored as an effective feature to promote the accuracies of predicting a β–turn [16], a γ–turn [17], a unified turn model [18], a DNA-binding residue [19] and a domain boundary [20]. The shape string was also considered as a backbone string to reconstruct the modeling of membrane proteins [21]. Accordingly, we constructed a web server, DSP [22], to predict the protein shape string from the sequences based on innovative technologies: a knowledge-driven sequence alignment and a sequence shape string profile.

Here we demonstrate a new approach, NMRDSP, which is an extension of DSP and can more accurately predict the protein shape string based on NMR CSs and structural profiles obtained from sequence data. A non-redundant set (1,003 entries) was explored as the training set of NMRDSP. Six NMR CSs (HA, H, N, CA, CB and C) were collected from the Biological Magnetic Resonance Bank (BMRB) database and were normalized and alphabetized. The structural profile of the residues was obtained from the DSP web server, which used the sequence data. The normalized and alphabetized NMR CSs and structural profiles were adopted as features (14 features) to input into a classification algorithm of conditional random field (CRF). The results confirm that the NMR CS and the structural profile are the significant features required for the prediction of the shape string and the combination of both of them significantly improves the accuracy of the predictor.

## Materials and Methods

### Data sets of chemical shifts and protein shape strings

All of the NMR CS data used in the NMRDSP were retrieved from the BMRB database [23] as of 2013. In the BMRB database, there were 6,670 entries, in which 4,036 sequences matched PDB sequences. We used the PISCES program [24] to reduce the redundancy of the 4,036 entries and determined that there were no two chains that had more than 25% sequence identity. In total, 1,381 entries were obtained which had both NMR CS data and three-dimensional structures. These entries were filtered further manually. We deleted the entries that had incomplete NMR CS data, incorrect branches, non-standard residues and any duplicates. Finally, 1,187 entries remained. As with TALOS+, six NMR CSs HA, H, N, CA, CB and C were used in NMRDSP.

We retrieved the shape strings of the sequences obtained from the above step from the website: http://www.fos.su.se/~pdbdna/ [12]. Due to the intrinsically disordered regions and sequence breaks, the sequences that had observed shape strings did not completely match the sequences that had NMR CS data, though they had the same PDB ID. A program was designed by C^#^ language to automatically align the residues in the sequences. However, there were several sequences that could not be completely aligned. We deleted such entries manually. In total, we obtained 1,003 entries, which had credible NMR CSs and observed shape strings. We named the set NS1003 and deposited these sequences and NMR CSs into the SHIFTY format [25]. The BMRB IDs and PDB IDs of NS1003 are listed in the Supplementary Materials S1.

The NS1003 set was divided into two subsets: NS203 and NS800. We randomly selected 203 entries from the NS1003 set and constructed an independent testing set. The rest of the entries were used as a crossover validation set to evaluate NMRDSP.

### Normalization and alphabetization of the NMR chemical shifts

The NMR CS data are pre-processed by normalization and alphabetization.

The NMR CS data are decimal real numbers, and different types of NMR CS have different distributions in different regions. In order to treat all types of NMR CS fairly we initially normalized the original data into [0, 1] regions using linear transformation. For a position in the NMR CS data that has been assigned vacancy, a tag “N” is labeled which is not counted in the linear transformation. The linear transformation formula is:



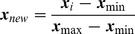
(1)


where, *x_new_* is the new value after linear transformation, *x_i_* is the original value of CS, *i* denotes one type of CS from one of the 20 common amino acids, *x_min_* is the minimum of one type of CSs from one amino acid and *x_max_* is the maximum of one type of CS from one amino acid. Therefore, there are 120 *x_min_* and 120 *x_max_* stored in NMRDSP for linear transformation.

The aim of linear transformation is to make the feature of each type of CS from each amino acid distribute in equal regions. Another aim is to prevent outliers. For one of six CS for an amino acid in a query, if it’s value is greater or less than the maximum or the minimum, respectively, for a special type of amino acid and a special type of CS, this value will be set as the maximum or minimum obtained from the training set.

**Figure 1:**
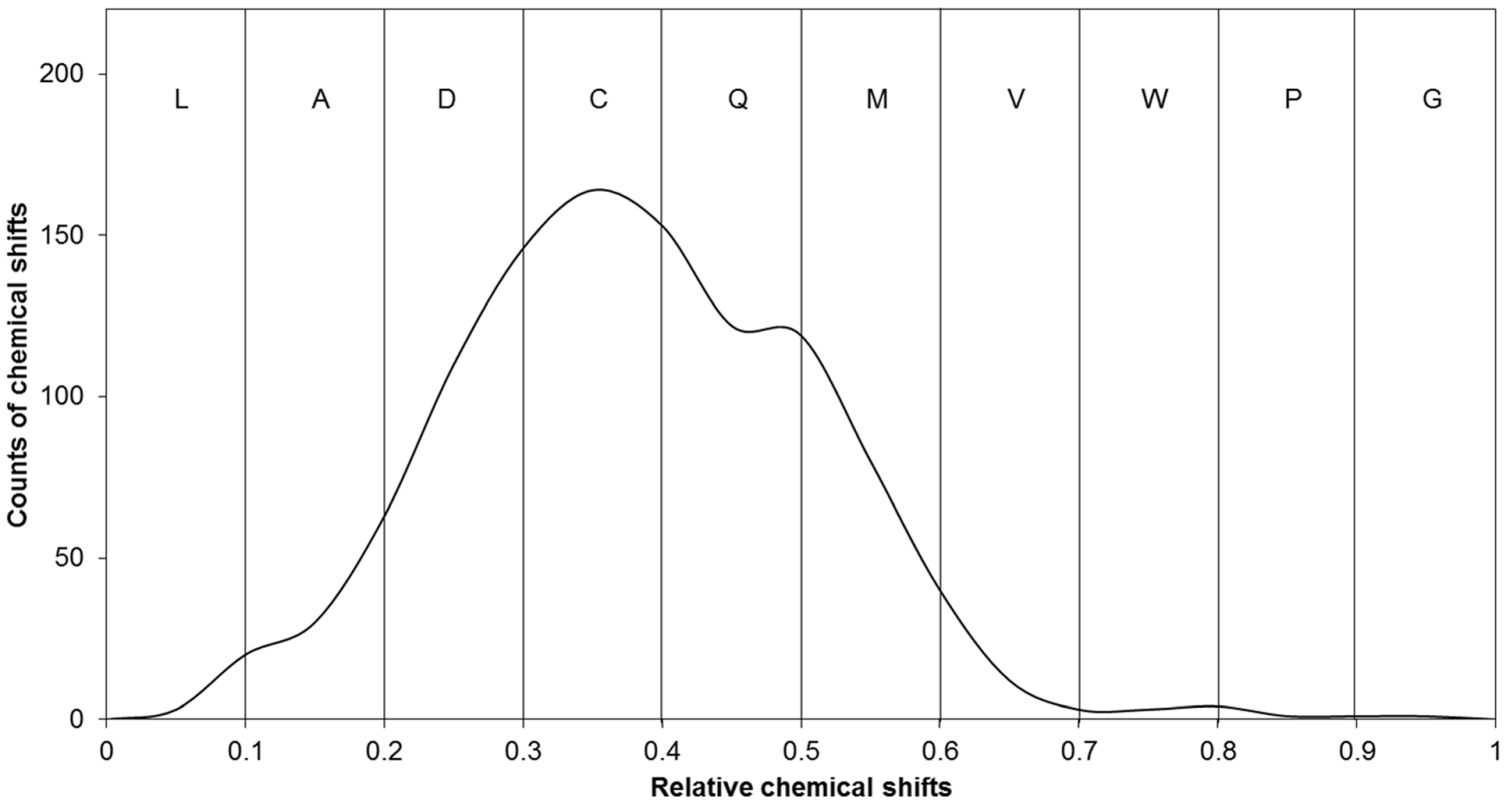
An example of normalization and alphabetization of Cystine C NMR CS data. After normalization, the values of NMR CS distribute from zero to one (horizontal ordinate). After alphabetization, each sub-region is expressed a character (top). The performances of pre-processing are given in Table 1.

These linear transformed values are then alphabetized. It is well known that NMR CS data are often affected by changes in environmental conditions: pH and temperature for example. Different environmental conditions cause slight shifts in NMR CSs. In order to tolerate these variations, we performed discretization of the NMR CSs. Each linear transformed region [0, 1], was divided into ten equal sub-regions (Figure 1). The NMR CS data that belonged to a sub-region were expressed by the same character in a string (L, A, D, C, Q, M, V, W, P and G). Adding “N” gave 11 letters for each of six NMR CS features that were used to express the NMR CS data. The alphabetized features can be manipulated by CRF.

### Shape string and its profile

In most cases, the backbone of a protein can be precisely described by the φ/ψ torsion angle pairs of the constituent amino acids. A shape string is defined as a classified region [12], [26] in the torsion angle space and is a way of coarse grained protein structural representation. There are eight characters (S, R, U, V, K, A, T and G) used to record shape states. There may be some positions of a query sequence that have no available shape string data. An empty position is expressed as “X”, wherein the shape string is represented by nine characters (A, S, K, R, T, U, V, G and X). Shape A represents *α*-helix and shape S represents *β*-sheet. Shape K is found at ends of helices or in 3_10_ helices. Shape R is the polyproline type II structure. The turn region is denoted as shape T. Shape U and V represent bridge regions. Shape G is special for glycine. Shape string is a one-dimensional string of symbols, which can carry more structural information than the classical secondary structure representation [27]. Typically, shape T reflects the turn structure in protein, and predicted shape T could help to identify the turns [16], [17]. The observed shape string can be freely obtained based on a sequence of known structure from the web server [12]. We have constructed a DSP web server to accurately predict the shape strings of protein sequences [22]. DSP has developed two innovative technologies: a knowledge-driven sequence alignment and a shape string profile strategy. For a query sequence, the outputs of DSP are predicted shape strings and shape string profiles.

In DSP, a hallmark pattern was defined as conservative in both the sequence patterns and the shape string structures. We initiated a traversal search for consecutive sequence patterns with sufficient frequency in a representative non-redundant PDB chain set (nr0PDB, NCBI MMDB 2009 Dec, 7,775 entries, 0-level non-redundancy). We developed an algorithm to extract candidate patterns from unequal length sequences without sequence alignment. The frequency criterion was set to 100 and 5,667 consecutive sequence patterns were obtained. For each position of a consecutive sequence pattern, the p-value of the corresponding shape string of the amino acid was calculated according to a binomially distributed model. Based on the p-values, we selected 2,761 hallmark patterns with lengths ranging between two and four residues that typically exhibited conserved structures to construct a hallmark pattern library. The hallmark pattern represented remote homology in the sequences and shape strings, and was an indispensable tool in generating the shape string profile.

The sequence shape string profile was generated as follows: In the first step, the query sequence was aligned using PSI-BLAST [28] against the nr3PDB (NCBI MMDB 2009 December, 3-level non-redundancy, 40,849 entries in total) resulting in the top N (default is 10) subjects. We utilized the hallmark patterns to hit the unmatched fragments and obtain the hit segments. These hit segments and their flanking amino acids (+S and -S, default is 5) were aligned together against nr3PDB using PHI-BLAST. The matched fragments obtained by the first alignment and the shorter sequences obtained by the subsequent alignments were counted and stored in eight boxes. Lastly, these boxes constituted a vector that represents the sequence shape string profile for each residue and was considered to include the structural hallmark pattern and shape string evolutionary information. The DSP is described in the Supplementary Materials S2. The shape string profile obtained from DSP is termed the DS_Profile in the following text.

### Sequence and secondary structure information

Sequence information is expressed by the position-specific scoring matrix (PSSM). PSSM is constructed from the multiple alignment of the top-scoring BLAST responses to a given query sequence [28] and is considered to contain evolutionary information of the sequence. PSSM is widely used as an effective feature to predict protein structure and function. Recently, SPSSM (structural position-specific scoring matrix) was proposed to improve the accuracy of the prediction of protein secondary structure [29]. SPSSM is a distinctive PSSM-like profile, which contains evolutionary information of protein secondary structure. A description of SPSSM is shown in the Supplementary Materials S3. PSSM and SPSSM were explored as selectable features in this study.

### Architecture of NMRDSP

**Figure 2:**
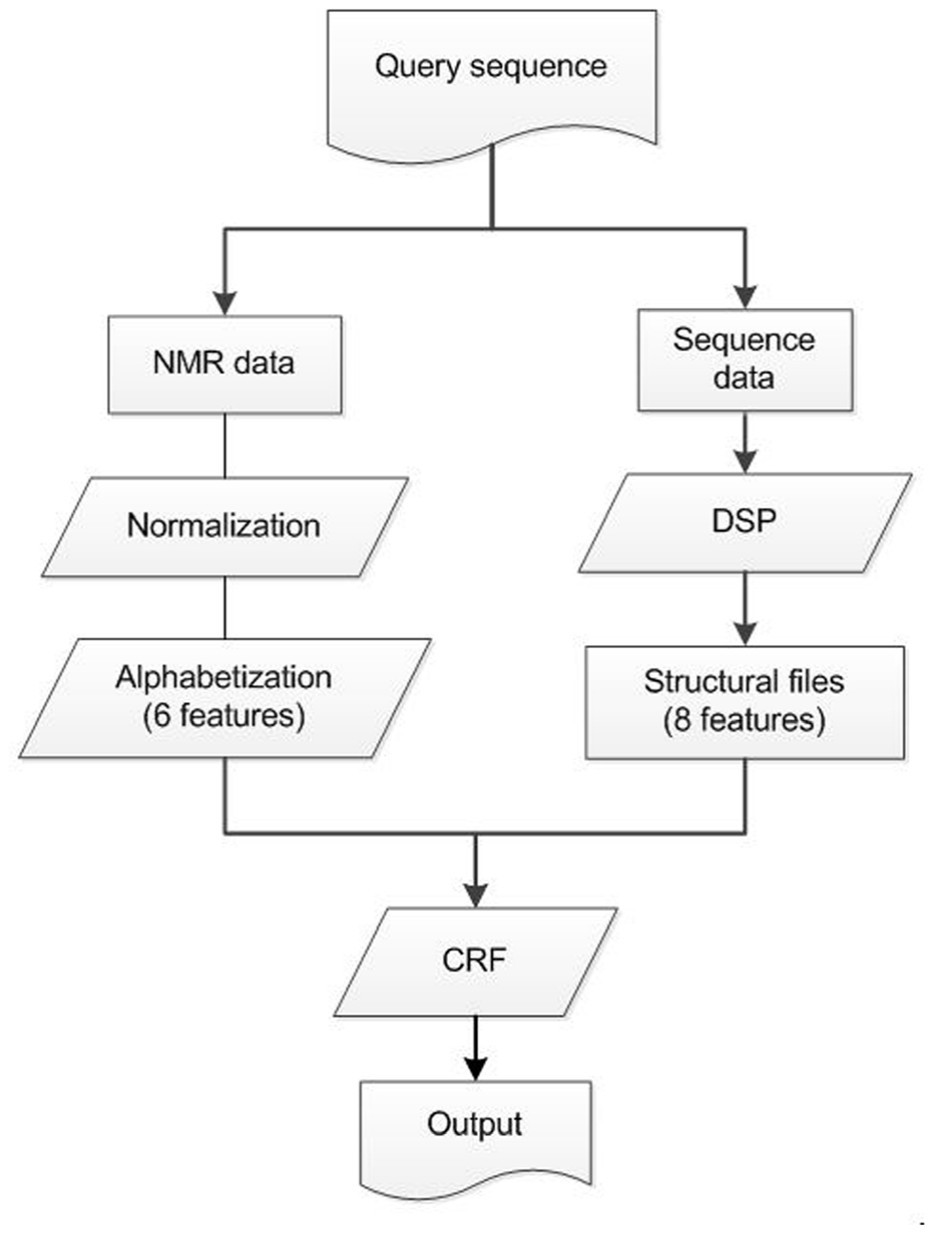
The flowchart of NMRDSP. There are four procedures in the flowchart. The normalization and the alphabetization are pre-processed of NMR CS data. The DSP is used to generate shape string profiles. Then 14 features are input for CRF.

The flowchart for NMRDSP is shown in Figure 2. For a submitted query, in SHIFTY format [25], NMRDSP extracts the query NMR CS data and the query sequence data. For the obtained NMR CS data, NMRDSP checks the availability of the data, and normalizes and alphabetizes the NMR CSs. There are six alphabet features for each amino acid in the query. For the obtained query sequence(s), DSP is performed to generate the shape string profiles. There are eight alphabet features for each amino acid in the query sequence. When training is performed, the 14 features of the training sequences are used as an input for the CRF training program to construct a prediction model. When testing is performed, the 14 features of a query sequence are used as an input of the CRF prediction program to predict the shape strings of the query based on the obtained trained model.

### Performance measures

We adopt two criteria to evaluate the prediction performance: accuracy (S8 and S3) and segment overlap measure (SOV). S8 is eight-state accuracy and is defined as,



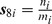
(2)


where *n_i_* is the number of correctly predicted the i shape string, *m_i_* is the total number of the i shape string. Eight-state shape string is mapped to the three states by [S, R, U, V]→S, [A,K] →H and [T,G] →T as defined by Zhou et al.[27]. S3 is a corresponding measurement and is calculated as a similar formula like the above.

SOV (Segment Overlap Measure) is a segment overlap measure and was defined by Zemla et al.[30], and has been selected as one of the predicted evaluation criteria. SOV is defined as,



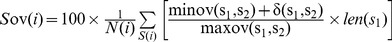
(3)


With the normalization value *N(i)* defined as:



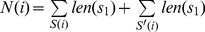
(4)




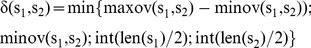
(5)


where, s_1_ and s_2_: The two secondary structure assignments being compared; len(s_1_): The number of residues in segment s_1_; minov(s_1_, s_2_) : The length of the actual overlap of s_1_ and s_2_; maxov(s_1_, s_2_): If both segments have residues in state *i,* the total extent for which either of the segments s_1_ and s_2_ has a residue in state *i.*

The expected value and its corresponding variance are determined by bootstrapping: 80% of the targets are randomly selected 1,000 times, and the average accuracy and the standard error of the scores are calculated [31].

## Results and Discussion

### Characteristics of NS1003

**Figure 3:**
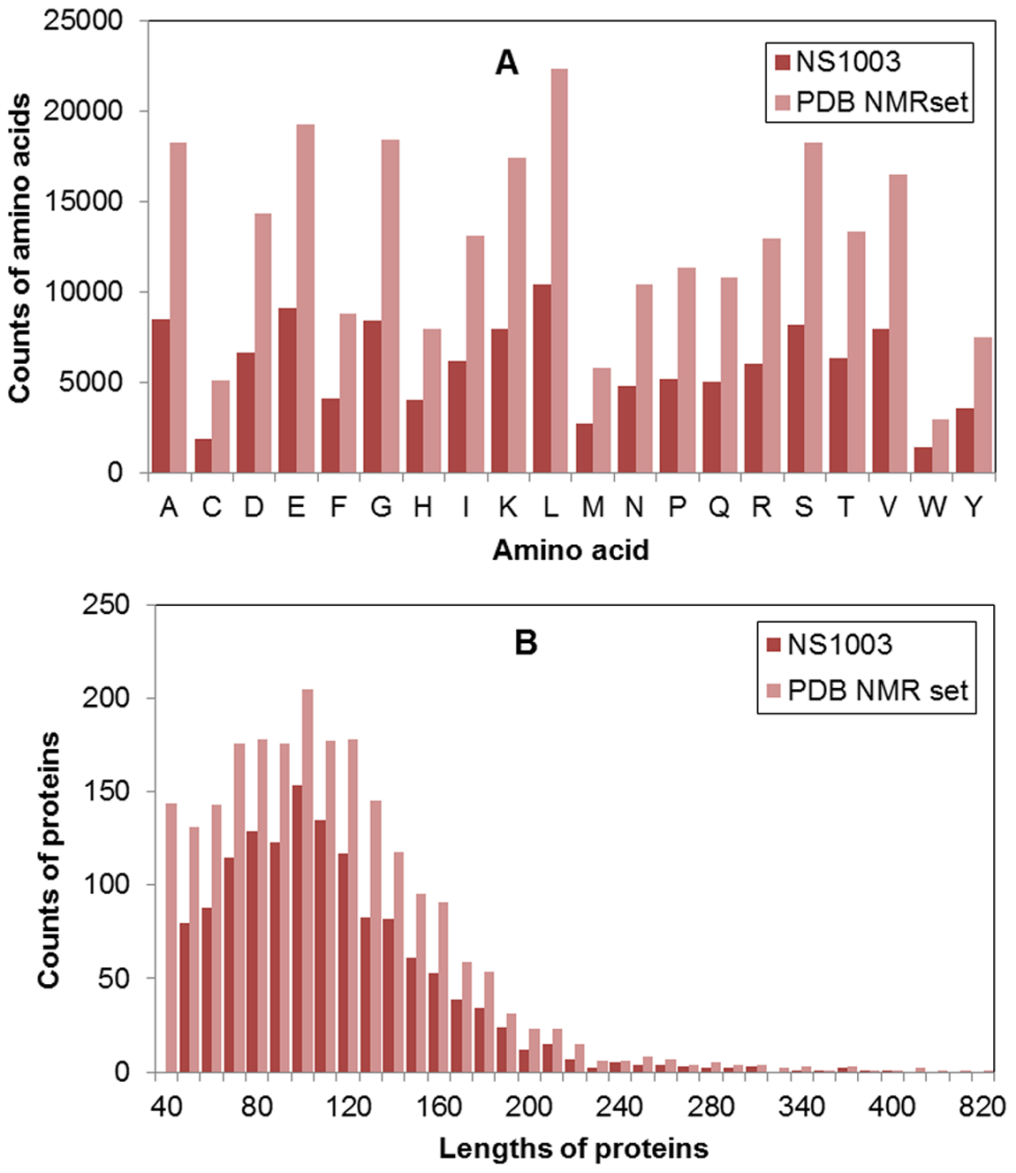
The characteristics of NS1003. (A) The numbers of amino acids in the NS1003 and the PDB NNMR set. (B) The distribution of the sequence lengths for NS1003 and PDB NMR set. These confirm that the non- redundant NS1003 set can represent the PDB NMR set.

NS1003 is a large set, which is compared with previous data sets and used in predictions of protein backbone conformations based on NMR CS. We analyzed the characteristics of NS1003. A comparison of frequency distributions of amino acids and distributions of sequence lengths are shown in Figure 3.

The PDB NMR set, shown in Figure 3, was collected from PDB as of 2013. All the structures of the sequences were determined by NMR experiments. These sequences had a reduced redundancy of 25%. There were 2222 entries in the PDB NMR set. Comparing the NS1003 with the PDB NMR sets, the distribution of the numbers of amino acids and the sequence lengths are very similar. This means that NS1003 is a good representative set of the PDB NMR data. There are a few sequences whose lengths are longer than 300 residues, limiting the region of study. We believe that NMR experiments will be able to relieve this bottleneck.

**Figure 4:**
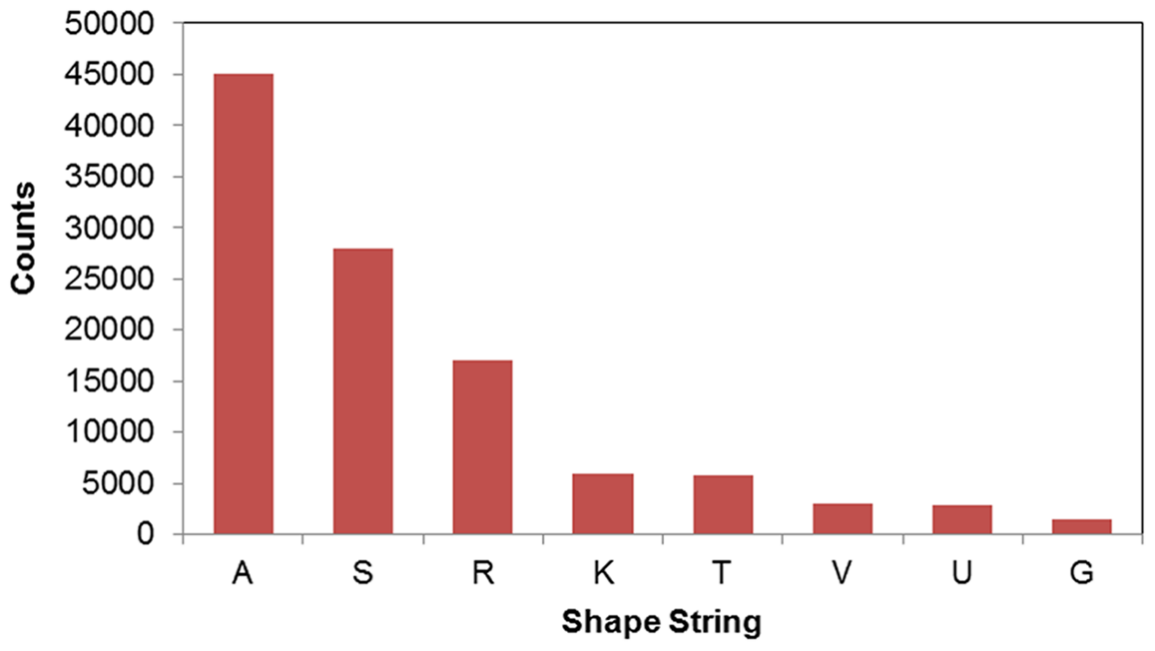
The distribution of the numbers of NS1003 residues in eight-state shape strings. The number of "A" strings is the most. The number of "G" strings is the least. The distribution is imbalance.

In the NS1003 set, there are 122,831 residues with defined shape strings. The distribution of the NS1003 residues in eight-state shape strings are shown in Figure 4.

The distribution of the numbers of residues in eight-state shape strings shows that the data is unbalanced in the shape string types. The number of shape string "A" is predominant. The number of "S" and "R" are in the middle of the range. This unbalanced data is a challenge to the multi-classification of the shape strings.

The distribution of normalized NMR CS data in NS1003 for amino acids is shown in the Supplementary Materials S4. The distributions of normalized NMR CS data in NS1003 for shape strings are shown in the Supplementary Materials S5.

### Performances of pre-processing

The pre-processing was performed on the NMR CS data. The original, normalized and alphabetized NMR CS data was used as the feature respectively. The results are shown in Table 1.

**Table 1:** Performances on NS1003 set by using the original NMR CS data, the normalized data and the alphabetized data (5-fold cross validation, %).

NMR data	S8	SOV8	S3	SOV3
Original	56.75±0.20	47.02±0.26	75.45±0.13	58.68±0.35
Normalization	61.18±0.23	53.04±0.27	78.42±0.20	66.95±0.35
Alphabetization	62.25±0.23	52.42±0.27	78.38±0.20	66.82±0.31

From Table 1, we can see the performance is improved after normalization, and the performance is slightly improved further on S8 after alphabetization. For other measurements (S3 and SOV), the differences appear in the regions of the variances, so the alphabetization of NMR CS is used in the NMRDSP.

### Selecting NMR chemical shifts

NMR CS is an easily obtained experimental datum. ^1^H, ^13^C and ^15^N data from proteins are available in several databases, including the BMRB database [23]. However, NMR CS data of a sequence are not always complete. This means there may be many CS positions of residues that are not recorded. For the positions that have not assigned effective CS values the letter "N" is used.

In NMRDSP only six NMR CS data, HA, H, N, CA, CB and C, were used as features. To determine the importance of these features, we used the leave one feature out for cross validation. Every NMR CS feature was removed one at a time and only once. It means five validations were carried out, and each used five features and removed a feature. The result is shown in Table 2.

**Table 2:** Performances of leave one feature out validations and using all six features on NS800 (5-fold cross validation, %).

Left feature	S8	SOV8	S3	SOV3
HA	61.41±0.24	51.04±0.28	77.80±0.21	65.71±0.33
H	61.71±0.24	51.29±0.29	77.56±0.19	65.26±0.36
N	61.84±0.23	51.91±0.29	77.76±0.20	65.88±0.34
CA	61.16±0.23	50.52±0.26	76.90±0.20	64.53±0.33
CB	61.88±0.24	51.85±0.26	78.13±0.21	66.42±0.33
C	62.02±0.25	52.17±0.27	78.2±0.22	66.45±0.31
Six features	62.25±0.23	52.42±0.27	78.38±0.20	66.82±0.31

In Table 2, all measurements are affected compared with using six NMR CS features (the last line in table 2) when a NMR CS feature is left out. The performance is the worst when the CA NMR CS is omitted. This illustrates that CA is the most important feature for prediction. According to the performances, we rank the importance as: CA>HA>H>N = CB>C. Although NMR CS "C" feature is at the end of the ranking, it still contributed 0.23%, 0.27%, 0.18% and 0.37% for S8, SOV8, S3 and SOV3 respectively. The results also correspond with the existed predictors, such as TALOS+ [8], in which all six NMR CS features were used. We decide to use all six NMR CSs features to predict shape string.

### Features of NMR CS, sequences and predicted structures

There are many reports to describe the prediction of protein backbone conformations using sequence and structural features. Selecting effective features is the key for successfully designing a protein structural sequence predictor. We summarize our experiments and find that an effective sequence and structure feature of a residue must be different when the residue appears in different surroundings. According to this rule there are several candidates of effective features: PSSM, secondary structure, solvent accessibility, shape string, sequence and structural motif. As a preparatory study, we explored the PSSM, SPSSM, NMR CS and DS_profiles as selectable features, which had been confirmed to be effective in prediction. The results are shown in Table 3.

**Table 3:** Performances of using PSSM, SPSSM, NMR CS and DS_Profile features on NS800 (5-fold cross validation, %).

Used feature	S8	SOV8	S3	SOV3
PSSM	50.96±0.22	40.43±0.23	65.62±0.19	48.27±0.28
SPSSM	56.17±0.26	43.24±0.28	70.84±0.24	56.07±0.32
NMR CS	62.25±0.23	52.42±0.27	78.38±0.20	66.82±0.31
DS_ Profile	71.7±0.29	64.06±0.37	82.03±0.25	71.53±0.40

The performances varied with features. As a feature of sequence the performance of PSSM is good. It makes the S3 accuracy of shape string prediction approximate to the Q3 accuracy of predicting secondary structure based only on sequence information. SPSSM gives an improved performance compared with PSSM. It confirms that the structural information is more useful for structural prediction. Undoubtedly, NMR CS has greatly improved the accuracy of shape string prediction in comparison with sequence and structural features. However, due to influences of environmental conditions, incorrect assignments and imperfections of NMR CSs, this performance is not perfect. It is clear that more effective features are expected.

The DS_Profile performs better than all of the other features tested. The benefit comes from the knowledge-driven sequence alignment. The DS_Profile is designed for predicting shape string [22] and it is not surprising that it is the critical feature for prediction.

### Combination of features

We assessed combinations of different features. The results are shown in Table 4.

**Table 4:** Performances of different feature combinations on NS800 (5-fold cross validation, %).

Feature Combinations	S8	SOV8	S3	SOV3
DS_Profile	71.7±0.29	64.06±0.37	82.03±0.25	71.53±0.40
DS_Profile+NMR	75.00±0.26	68.16±0.51	86.91±0.18	78.57±0.54
DS_Profile+NMR+SPSSM	74.87±0.26	68.01±0.51	86.95±0.17	78.36±0.54
DS_Profile+NMR+SPSSM+PSSM	73.17±0.28	66.28±0.46	86.02±0.19	76.23±0.47

Research was initiated using the DS_Profile feature, and other features were added successively. The best feature combination was using the DS_Profile and NMR CS. Using these two types of features NMRDSP achieved accuracy of 75% for S8 and 86.9% for S3. Adding PSSM and SPSSM did not improve the performance of prediction. The results illustrate that using the DS_Profile and NMR is the best performance for predicting shape string in the experiments of different feature combinations.

### Performance on the independent testing set

NS203 was used as an independent testing set to validate our approach based on the training set of NS800 and the features DS_Profile and NMR CS. The performance is shown in Table 5. We achieved an accuracy of 75.8% for S8 and 87.8% for S3.

**Table 5:** Performances of NS203 independent testing set (%) based on NS800 training set.

8-State	Accuracy	SOV	3-State	Accuracy	SOV
**S**	81.58±0.52	71.06±0.69	**S**		
**R**	60.72±0.81	58.17±0.90	**S**		
**U**	35.71±1.08	35.48±1.31	**S**		
**V**	32.55±1.20	33.71±1.39	**S**	87.90±0.31	80.47±0.55
**K**	38.19±1.04	37.76±1.14	**H**		
**A**	91.85±0.24	82.33±0.59	**H**	91.7±0.22	83.59±0.50
**T**	61.45±0.86	59.33±1.02	**T**		
**G**	43.13±1.50	43.02±1.79	**T**	60.61±0.76	59.39±0.96
**Total**	75.80±0.29	69.16±0.53	**Total**	87.81±0.23	80.58±0.43

From table 5 we can see that the predicted accuracies are different for different shape string types. For the largest "A" type (figure 4), accuracy achieves 91.8%, which is the highest accuracy comparing with other shape string types. On the other hand, due to the numbers of "V", "U" and "G" are less than the numbers of other types, their predicted accuracies are less than 40%. The imbalance affects the performances of multi-class classification.

**Figure 5:**
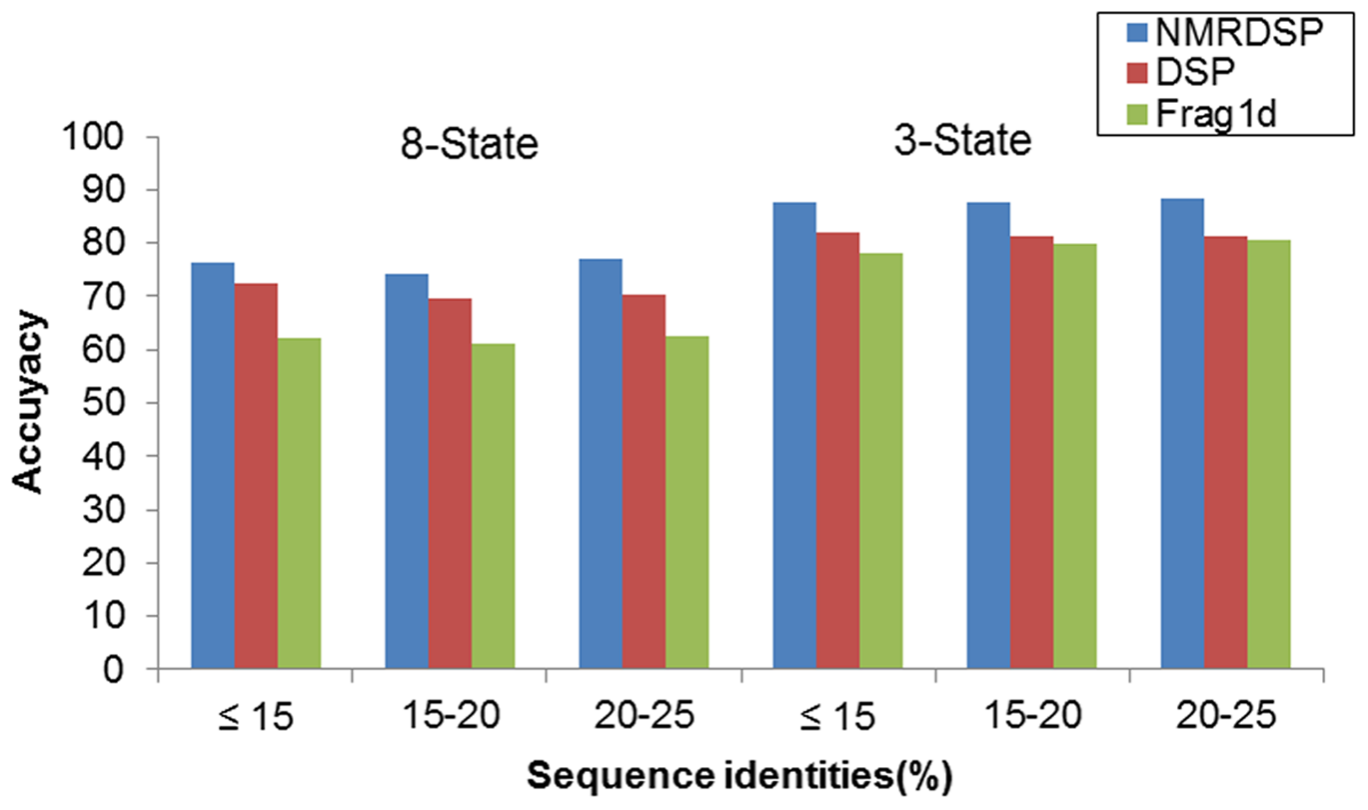
Performances of NMRDSP, DSP and Frag1D for three classes of different sequence identities.

The other element that affects the performances of a prediction is the sequence identity between the query and training set. The sequence identity between the query and training set is the foundation of machine learning approach. However, if the sequence identity between the training and the testing is high, it will cause over-estimation. If the sequence identity between the training and the testing is very low, the prediction will be near random guess. Usually, the sequence identity is measured by distance (according to the definition) between sequence pairs in sequence space. A robust approach should perform well when the sequence identity is low, for example less than 25%. The histograms of the pairwise sequence identities of NS800 and NS203 are given in Supplementary Materials S6. The independent testing set was divided into three classes according to pair sequence identities. The performances of three classes are showed in figure 5.

When the sequence identity is not greater than 15%, the S8 accuracy of NMRDSP achieved 76.1%, and the S3 accuracy achieved 87.6%. When the sequence identity is between 20% and 25%, the S8 accuracy of NMRDSP achieved 76.9%, and the S3 accuracy achieved 88.4%.

We compared our approach with DSP and Frag1D [25] on the independent testing set. The results are shown in table 6 and figure 5.

**Table 6:** A comparison of performances of NS203 by NMRDSP, DSP and Frag1D (%).

Method	S8	SOV8	S3	SOV3
**NMRDSP**	75.80	69.16	87.81	80.58
**DSP**	71.38	63.73	80.44	71.48
**Frag1D**	61.87	53.90	78.82	69.64

The NMRDSP had an improvement of accuracy (S8) of 4.4% and 13.9% compared with DSP and Frag1D respectively. It is indisputable that using NMR CS data can effectively improve the performance of shape string prediction.

The improvements on accuracies of performances mainly come from the novel technology: hallmark pattern. Hallmark pattern was defined as a short fragment that is conservative in both sequence patterns and shape string structures and could extract remote homology [22].

### NMRDSP web server

The NMRDSP web server was constructed according to Figure 2 and is freely available at http://cal.tongji.edu.cn/NMRDSP/index.jsp. The software of CRF is CRF++0.54 which is available at http://crfpp.sourceforge.net/. The training set was NB1003. The input file format of NMRDSP web server is SHIFTY. The template file of CRF and the input file format of NMRDSP are given in the Supplementary Materials S7. The software takes about one minute to analyze and process a query sequence. The output of NMRDSP is a downloadable text file which contains the query sequence(s), predicted shape strings and their probabilities.

## Conclusion

In this study we have demonstrated that NMR CS and the structural profile are significant features for predicting shape strings, and a combination of both has increased the accuracy of prediction. The NMRDSP web server has been constructed for shape string prediction. We believe NMRDSP could be employed as a solid platform to predict other protein structures and functions.

## Supporting Information

Supplementary Materials S1 **The corresponding relation between PDB ID and BMRB ID.**
(DOC)

Supplementary Materials S2 **DSP web server.**
(DOC)

Supplementary Materials S3 **Structural Position-Specific Scoring Matrix.**
(DOC)

Supplementary Materials S4 **The distributions of NMR after normalization for residues.**
(DOC)

Supplementary Materials S5 **The distributions of NMR CS data after normalization for shape strings.**
(DOC)

Supplementary Materials S6 **The sequence identities in NS800 and NS203.**
(DOC)

Supplementary Materials S7 **The CRFs Template and SHIFTY format.**
(DOC)
