# Supplementary material for: NMRDSP: An Accurate Prediction of Protein Shape Strings from NMR Chemical Shifts and Sequence Data
Source: PLoS One. 2013 Dec 23;8(12):e83532. doi: 10.1371/journal.pone.0083532 (PMC3871590; doi:10.1371/journal.pone.0083532)
Supplement: Supplementary Materials S1 — The corresponding relation between PDB ID and BMRB ID. (DOC) [file pone.0083532.s001.doc]

**Supplementary materials**

**S1. The corresponding relationship between PDB ID and BMRB ID**

**Table S1.1 Relationship between PDB ID and BMRB ID (Training Set)**

| **PDB ID** | **BMRB ID** | **PDB ID** | **BMRB ID** | **PDB ID** | **BMRB ID** | **PDB ID** | **BMRB ID** |
| --- | --- | --- | --- | --- | --- | --- | --- |
| 1JBIA | 5047 | 1G6EA | 4833 | 1NZPA | 5766 | 2HGKA | 15327 |
| 1BR0A | 4198 | 2JMPA | 15055 | 2LB5A | 17546 | 2KF2A | 16881 |
| 1GH9A | 4740 | 2L5PA | 17279 | 2JSAA | 15358 | 2GJIA | 7099 |
| 2KFVA | 16189 | 2CKAA | 15098 | 1Z4HA | 6762 | 2KVOA | 16782 |
| 2LC9A | 17603 | 2K2EA | 15702 | 2KOKA | 16517 | 1DS9A | 4265 |
| 2AGAA | 6742 | 1ZTSA | 6366 | 2KF4A | 16170 | 2KM4A | 16411 |
| 2BZTA | 6776 | 2KT9A | 16691 | 2LCKA | 17614 | 1WYOA | 11086 |
| 2KL5A | 16384 | 1FEXA | 4639 | 2K2XA | 15729 | 2AVXA | 6454 |
| 1XS8A | 6359 | 2KBZA | 16057 | 2GO9A | 7070 | 1V9VA | 10079 |
| 2KC6A | 16062 | 2DHYA | 11276 | 2KA5A | 16019 | 1L3XA | 5346 |
| 1LR1A | 5390 | 2KEPA | 16164 | 2RNNA | 11029 | 2DK4A | 11355 |
| 2KMVA | 16440 | 2IZ3A | 15037 | 2RPZA | 11061 | 2JRFA | 15329 |
| 1J9IA | 4752 | 1RW2A | 5907 | 2LGXA | 17827 | 1VGHA | 5238 |
| 2KNAA | 16478 | 1DV5A | 4603 | 2KFWA | 16240 | 1OP4A | 5786 |
| 2KBIA | 16045 | 2G7JA | 7063 | 1D4BA | 4574 | 2KKVA | 16373 |
| 2LDKA | 17670 | 1NYNA | 5695 | 2KK4A | 16352 | 2DATA | 11329 |
| 2KYWA | 16988 | 2FJ6A | 6976 | 2KMWA | 16442 | 1YFBA | 6478 |
| 2EE2A | 10270 | 1X3AA | 11286 | 2L7EA | 17352 | 1C05A | 4577 |
| 1Z2FA | 6111 | 1FZTA | 4648 | 1MJDA | 5482 | 2FE0A | 6910 |
| 1KN7A | 5236 | 2L4OA | 17246 | 2KYRA | 16979 | 2K50A | 15819 |
| 2E0HA | 7330 | 2LD7A | 17653 | 2KISA | 16293 | 1Y6UA | 6479 |
| 2K01A | 15633 | 2K1HA | 15678 | 2YH0A | 17622 | 1CE3A | 4487 |
| 2D1UA | 6803 | 2KS0A | 16656 | 1JAJA | 5010 | 1R73A | 5977 |
| 2PP4A | 7396 | 2GT3A | 6090 | 2L6NA | 17318 | 2KZ6A | 16999 |
| 2HWTA | 7112 | 2BICA | 6520 | 2KMAA | 15615 | 2JXUA | 15574 |
| 1S6NA | 6046 | 2LD6A | 17651 | 2COMA | 10011 | 1KG1A | 5199 |
| 2RN7A | 11017 | 2KYYA | 16991 | 2JNGA | 15109 | 2F63A | 4299 |
| 2BW2A | 6731 | 1XC5A | 6286 | 1SS3A | 6139 | 1N91A | 5596 |
| 2JMLA | 15023 | 2JNSA | 15125 | 2LA7A | 17509 | 2CTKA | 11136 |
| 2HA1A | 7128 | 2E0GA | 10027 | 2L3TA | 17202 | 2OIIA | 5882 |
| 2L3BA | 17176 | 1K0XA | 5220 | 2JZBA | 15614 | 1M4PA | 5532 |
| 1TXEA | 6254 | 2PXGA | 6797 | 1WYPA | 11124 | 2JOEA | 15167 |
| 1WM4A | 6032 | 2RRUA | 11443 | 2LF3A | 17737 | 2KKUA | 16372 |
| 2E6JA | 11091 | 2JS5A | 15354 | 2KX2A | 16897 | 2JNZA | 15134 |
| 1OM2A | 4496 | 2K6WA | 15894 | 2JX5A | 15547 | 1GO5A | 5364 |
| 1KMDA | 5366 | 1KLQA | 5299 | 1RW5A | 5599 | 1EZYA | 4386 |
| 2CH0A | 6919 | 2KSRA | 16672 | 2G0QA | 7007 | 1Q27A | 5570 |
| 2KJLA | 16339 | 1JCUA | 5051 | 1SSNA | 4215 | 2DHSA | 6121 |
| 2K1GA | 15603 | 2L5EA | 17270 | 2KUTA | 16746 | 2K2PA | 15721 |
| 2JOOA | 15405 | 2K42A | 16824 | 1RRZA | 6108 | 2JSWA | 15411 |
| 2L8NA | 17419 | 1LM0A | 5407 | 2DAWA | 11294 | 2C52B | 6874 |
| 2LFEA | 17750 | 2KK7A | 16354 | 2KL7A | 16386 | 2FI2A | 6957 |
| 2LC2A | 17588 | 2L8KA | 16977 | 1J0TA | 5633 | 1SJ6A | 6152 |
| 1WFOA | 10076 | 2I9YA | 7339 | 1JOOA | 4052 | 2KY5A | 16935 |
| 2DI0A | 11278 | 2KZ4A | 16997 | 1RZSA | 6185 | 1WJKA | 10060 |
| 2JS7A | 15356 | 2JQ5A | 15270 | 2AQAA | 6846 | 1XSFA | 6221 |
| 2IF1A | 4255 | 1HA8A | 4979 | 2LFKA | 17763 | 2Y9TA | 17475 |
| 2DZLA | 11282 | 2DDJA | 6963 | 2J4NA | 7285 | 2KCUA | 16097 |
| 2L5VA | 17286 | 2KCNA | 16087 | 2KBBA | 15457 | 2K4NA | 15805 |
| 2KDDA | 16110 | 1Q6AA | 5824 | 2GRIA | 7019 | 1XQ8A | 5744 |
| 1H9FA | 5043 | 1H95A | 4147 | 2IVWA | 7209 | 2KDOA | 16119 |
| 1T0YA | 6176 | 2K5IA | 15837 | 2JN8A | 15089 | 2JW8A | 15511 |
| 2KN6A | 15313 | 2KVTA | 16791 | 2YRQA | 11147 | 1WWYA | 10336 |
| 1YDUA | 6443 | 1H3ZA | 5538 | 1HBWA | 5180 | 1CKVA | 4431 |
| 2LD4A | 17646 | 1I4VA | 5022 | 2K73A | 15966 | 1SZLA | 6175 |
| 1U89A | 6285 | 1XPNA | 6343 | 2KFBA | 16173 | 2L2DA | 17132 |
| 2K9PA | 15995 | 2KLBA | 16388 | 1JDQA | 5060 | 2ORCA | 4207 |
| 2KULA | 16738 | 2JVWA | 15491 | 2KTLA | 16832 | 1R36A | 5991 |
| 2CU7A | 11322 | 2JRPA | 15338 | 1RXLA | 5947 | 2KR9A | 16632 |
| 2L4EA | 17238 | 2L82A | 17390 | 2JZAA | 15611 | 2KKNA | 16366 |
| 1IRZA | 5174 | 2K75A | 15902 | 2GZOA | 7180 | 1WXAA | 11264 |
| 2L9MA | 17478 | 2HAJA | 6284 | 2JPDA | 15240 | 2KEYA | 16161 |
| 2L5CA | 17272 | 2YSZA | 10237 | 2HJQA | 7201 | 1L6TA | 5326 |
| 2JY9A | 15584 | 1F3CA | 4912 | 2JNKA | 7269 | 1IN1A | 5041 |
| 2I83A | 5903 | 2JOQA | 15190 | 2HUGA | 7241 | 1XWEA | 6001 |
| 2KYSA | 16981 | 2JWYA | 15542 | 2K8VA | 7430 | 2L7KA | 17359 |
| 1RI9A | 5467 | 2L06A | 17031 | 1Z1MA | 6612 | 1CEJA | 4437 |
| 1WGNA | 11260 | 1L8YA | 5392 | 1YVCA | 6574 | 2JXWA | 15575 |
| 2EXDA | 10118 | 1POQA | 5836 | 2V1NA | 6938 | 2L02A | 17026 |
| 1ZG2A | 6717 | 2KMUA | 16544 | 2L6OA | 17320 | 2KJFA | 16319 |
| 2OQ3A | 15126 | 2FEKA | 6934 | 1V5SA | 10026 | 1NY4A | 5691 |
| 2L3FA | 17191 | 2LG1A | 16195 | 1WH5A | 10044 | 2GUTA | 7185 |
| 2K4QA | 15807 | 1HDLA | 4910 | 2RR8A | 11175 | 2A05A | 6740 |
| 2YRPA | 11148 | 3NLAA | 4199 | 1QTSA | 6034 | 2KEAA | 16141 |
| 2JXPA | 15568 | 1T3VA | 6198 | 2DOGA | 10138 | 1O6XA | 5561 |
| 2O4EA | 7270 | 2YS4A | 11090 | 1S6DA | 6082 | 2HI6A | 7228 |
| 2JMSA | 15058 | 2L57A | 17267 | 2L1CA | 17080 | 2K5RA | 15847 |
| 1TM9A | 6244 | 1PVEA | 5868 | 1X37A | 6615 | 1XFNA | 6321 |
| 2KFPA | 16186 | 2KELA | 16153 | 2BZEA | 7351 | 1UHUA | 5880 |
| 1ZZAA | 6715 | 2KW8A | 16811 | 2KLYA | 16404 | 1JYTA | 4735 |
| 1M9WA | 5475 | 2KWHA | 15524 | 2LLGA | 18047 | 1V63A | 10111 |
| 2KPJA | 16557 | 1MM4A | 5557 | 2OFNA | 15038 | 2KK1A | 16349 |
| 2EKHA | 10272 | 1Z00B | 6551 | 2DAZA | 11187 | 2HG7A | 7224 |
| 2KZWA | 17021 | 2KRUA | 16649 | 1SQ8A | 6084 | 2JRAA | 15324 |
| 1CX1A | 4706 | 2KZ5A | 16998 | 2KCQA | 16093 | 1JJRA | 4941 |
| 2DZJA | 11280 | 2JPNA | 15252 | 2K8SA | 15347 | 2KLLA | 16317 |
| 2K48A | 15790 | 1I1SA | 4957 | 2KL8A | 16387 | 2DAVA | 11212 |
| 2LE1A | 17688 | 2K2JA | 15707 | 2K7QA | 15925 | 1Q8KA | 5917 |
| 2KJVA | 16344 | 2K6AA | 15863 | 1MO7A | 5577 | 2K85A | 15938 |
| 1L1IA | 5323 | 2GVSA | 7184 | 2KC0A | 16058 | 2RRDA | 11252 |
| 1UGLA | 5848 | 2GI4A | 7189 | 2JSYA | 15798 | 2RREA | 11250 |
| 2KRXA | 16652 | 2FINA | 6809 | 1K9CA | 5204 | 2KIVA | 16297 |
| 2KG4A | 15855 | 2KQPA | 16608 | 1JFNA | 5075 | 2H7AA | 7182 |
| 1R3BA | 5833 | 2YS0A | 11150 | 2KNRA | 16476 | 1N4CA | 4716 |
| 2KWPA | 16868 | 2JMKA | 15039 | 2JSOA | 15371 | 2YT2A | 11095 |
| 2L6QA | 17321 | 2L04A | 17028 | 1WFYA | 10056 | 2LBUA | 11064 |
| 1X1FA | 10250 | 1Q9PA | 5967 | 2KYMA | 16970 | 2DA4A | 10286 |
| 1N89A | 4977 | 1WFSA | 10055 | 2K4VA | 15810 | 1HOYA | 4948 |
| 2JZCA | 15617 | 1MFNA | 4206 | 2KA7A | 16016 | 2JVVA | 15490 |
| 2X8NA | 16790 | 2K8AA | 16818 | 2KVRA | 16789 | 2KIWA | 16298 |
| 2KW5A | 16806 | 2K7NA | 15922 | 1SSFA | 5878 | 2HSTA | 6763 |
| 1UJXA | 10104 | 2L2QA | 17150 | 2AHQA | 6816 | 2JQQA | 15290 |
| 2L3NA | 17199 | 2JTYA | 15423 | 2L3AA | 17175 | 2LE2A | 17689 |
| 1OQKA | 5710 | 1NXIA | 5589 | 2ROPA | 11041 | 2DA7A | 10289 |
| 2K2OA | 15718 | 2KWUA | 16880 | 2LAHA | 17524 | 2KN8A | 16452 |
| 2JZDA | 15618 | 2RPBA | 11060 | 2K4JA | 15801 | 2HDLA | 7229 |
| 2KLZA | 16405 | 1WK1A | 10035 | 2KERA | 16157 | 2JQEA | 15275 |
| 1XRDA | 6349 | 2G31A | 7067 | 2LDYA | 17686 | 2KYGA | 16954 |
| 1XDXA | 4929 | 2LBSB | 17574 | 1NWVA | 5506 | 2AJ0A | 6811 |
| 2DAFA | 11270 | 2K7ZA | 15932 | 2P6JA | 7401 | 2KPQA | 16564 |
| 2KCJA | 16082 | 2A9HA | 6728 | 2KIJA | 16277 | 1F2HA | 4636 |
| 2DMEA | 11334 | 1XSAA | 6330 | 2AL3A | 6761 | 1WFQA | 10054 |
| 1G03A | 4649 | 1UFMA | 5849 | 2KNCA | 16496 | 1AKEA | 5720 |
| 2JR0A | 15312 | 1N5PA | 5688 | 1NNVA | 5779 | 2JS4A | 15353 |
| 2KZSA | 17018 | 2KHKA | 16247 | 1WKTA | 5255 | 2D87A | 11142 |
| 2KCWA | 16098 | 2LCYA | 17638 | 2LBBA | 17553 | 1RQUA | 4429 |
| 2G0UA | 6981 | 2RQ1A | 11065 | 1GO0A | 5485 | 2KUDA | 16732 |
| 1WHUA | 10127 | 1SAPA | 5905 | 2I3EA | 7167 | 1YELA | 6464 |
| 2A4HA | 6738 | 2KZXA | 17022 | 2RRLA | 11423 | 2K0QA | 15655 |
| 1U3NA | 6073 | 1VDIA | 10012 | 1M2EA | 5031 | 2L55A | 17266 |
| 2KSVA | 16675 | 1P6RA | 5873 | 1OVYA | 5970 | 1WF9A | 10130 |
| 2LBOA | 16233 | 2JQOA | 15288 | 2KXIA | 16737 | 1WJJA | 10090 |
| 2KSFA | 16922 | 2CSPA | 11183 | 2B87A | 6806 | 1COUA | 4396 |
| 1KN6A | 5242 | 2KMDA | 16426 | 1B75A | 4395 | 2JOHA | 15394 |
| 2KA0A | 16007 | 1XU6A | 6419 | 2KT7A | 16686 | 2C0SA | 7349 |
| 2KW7A | 16810 | 2KZ9A | 17000 | 2L81A | 17389 | 2KW6A | 16808 |
| 2FQHA | 6812 | 1G5VA | 4899 | 1LS4A | 4814 | 2K27A | 15693 |
| 2HFIA | 7227 | 2LF2A | 17736 | 2L4BA | 17235 | 2KCZA | 16100 |
| 1WINA | 10059 | 1KVVA | 4935 | 2RMKA | 11010 | 2HH8A | 7274 |
| 1T6WA | 6201 | 1JBJA | 5072 | 2KC5A | 16061 | 1OQAA | 6114 |
| 2COOA | 11240 | 2JUBA | 15438 | 1ICHA | 5018 | 2K54A | 15823 |
| 2K4TA | 16381 | 1J8IA | 5042 | 1GHTA | 4269 | 2RM4A | 11009 |
| 2D9ZA | 10312 | 1WGRA | 10040 | 2AKKA | 6746 | 1RY3A | 6069 |
| 2JPQA | 15258 | 2KNGA | 16831 | 2K37A | 15742 | 2JPUA | 15265 |
| 1NEIA | 5621 | 1TDPA | 6211 | 1K8OA | 5078 | 2DAEA | 11269 |
| 2RPJA | 11346 | 2I7UA | 15021 | 2DKQA | 10318 | 2JT1A | 15386 |
| 2AIHA | 7078 | 2A7YA | 7000 | 1UFGA | 10120 | 2L9FA | 17355 |
| 1KRIA | 5275 | 2KSYA | 16678 | 1LQ7A | 5356 | 1RQ6A | 6028 |
| 2L3RA | 17200 | 2KPUA | 16570 | 2GYTA | 7120 | 1TUJA | 5030 |
| 2BBUA | 6580 | 2KC8A | 16065 | 2K9NA | 15989 | 1XO8A | 6339 |
| 2KPYA | 16111 | 1SM7A | 6137 | 2G1DA | 6989 | 2A2YA | 5892 |
| 2LCJA | 17418 | 2JTMA | 15415 | 1WHRA | 10126 | 2K6IA | 15870 |
| 1KTUA | 5261 | 1X60A | 6649 | 2KCDA | 16072 | 2DJRA | 10257 |
| 2JSSA | 15393 | 2AI6A | 6625 | 2JZ6A | 15609 | 2K1MA | 15679 |
| 2L9BB | 17161 | 2IN2A | 5659 | 1Z5FA | 5787 | 2RNOA | 11030 |
| 1NQ4A | 5664 | 2JUGA | 15442 | 2Z4FA | 15315 | 1V5OA | 11253 |
| 2EZHA | 4090 | 2EE5A | 10145 | 2DJVA | 10261 | 1JI8A | 5115 |
| 1T1HA | 6265 | 2V9HA | 15247 | 2LA3A | 17501 | 2KZRA | 17017 |
| 1R9KA | 5701 | 2KTAA | 16692 | 2KDIA | 16114 | 2DAMA | 11274 |
| 1TQZA | 6354 | 2JR1A | 15314 | 2JSNA | 15370 | 1BHUA | 4217 |
| 2JRMA | 15339 | 2ROHA | 11038 | 1TEYA | 6298 | 1NR3A | 5657 |
| 1NY8A | 5798 | 2L9DA | 17448 | 1LS8A | 6313 | 1SB6A | 6172 |
| 2JOPA | 15189 | 2K1SA | 15683 | 2K5CA | 15828 | 2AYXA | 6810 |
| 1NG7A | 5753 | 2KVVA | 16795 | 1R05A | 5956 | 2L8TA | 17425 |
| 2KXYA | 16927 | 2BVBA | 6376 | 2L7BA | 15744 | 1PJZA | 5820 |
| 2JQFR | 15277 | 1KTMA | 5266 | 2K43A | 15783 | 2K9QA | 15999 |
| 1JR6A | 4791 | 2NRGA | 7320 | 2KO1A | 16486 | 2LHFA | 17842 |
| 1IJCA | 5097 | 2KZBA | 17006 | 2C34A | 6794 | 2OUTA | 6780 |
| 1Q8GA | 6004 | 2ARWA | 5940 | 2JZ2A | 15604 | 2DBAA | 11122 |
| 2JQ3A | 15268 | 1L6NA | 5316 | 2FS1A | 6945 | 2K8EA | 15943 |
| 2WCYA | 15996 | 2CZNA | 6829 | 2ADNA | 6743 | 2KRRA | 16646 |
| 2JZ4A | 15607 | 1S6LA | 6047 | 2LC0A | 17585 | 1V95A | 11255 |
| 2NPBA | 7324 | 2K5PA | 15844 | 1JW2A | 5166 | 2TMPA | 4214 |
| 2LJPA | 17952 | 2EKIA | 10273 | 2KXEA | 16986 | 2GZPA | 7178 |
| 2L4WA | 17257 | 1USTA | 6161 | 2XK0A | 17050 | 2K86A | 15939 |
| 2KOUA | 16534 | 1BLRA | 4186 | 2L1NA | 17090 | 2KSHA | 16662 |
| 2KRAA | 16633 | 1XOXA | 6342 | 2HEPA | 7225 | 2L4VA | 17258 |
| 2BIDA | 5340 | 1IW4A | 5348 | 2RNJA | 11024 | 2DALA | 11273 |
| 2LHSA | 17160 | 2LJUA | 17962 | 2KD7A | 16107 | 2GQBA | 7079 |
| 2KYQA | 16978 | 1X5BA | 10264 | 2K29A | 15691 | 2K19A | 15673 |
| 1J7MA | 5012 | 1NH4A | 5815 | 1SE9A | 6128 | 2KZAA | 17001 |
| 1UVGA | 6180 | 2LAGA | 16677 | 2KICA | 16269 | 1WLNA | 11246 |
| 2KUMA | 16839 | 2KK2A | 16350 | 2L0WA | 17066 | 1U5MA | 6299 |
| 2K0MA | 15652 | 1UJSA | 10069 | 2KL3A | 16382 | 2K9IA | 15987 |
| 2C06A | 6925 | 2BAFA | 6893 | 1S04A | 6045 | 2HGCA | 7260 |
| 1Y7XA | 6447 | 2LGRA | 15593 | 1XPWA | 6344 | 2L1IA | 17085 |
| 2L1SA | 17104 | 2RRFA | 11251 | 2KRTA | 16648 | 1Q5FA | 5879 |
| 2L6UA | 17323 | 1Z87A | 6752 | 2AFJA | 6311 | 1BUQA | 4230 |
| 2JN0A | 15079 | 2E7MA | 11232 | 2KSDA | 16947 | 1K5OA | 5175 |
| 2KSWA | 16676 | 2QMVA | 6549 | 1JJGA | 5077 | 1WI0A | 10046 |
| 2B3AA | 6894 | 1X0OA | 6597 | 1Q53A | 5843 | 1WJWA | 11310 |
| 2KFDA | 16176 | 2RRNA | 11426 | 1K45A | 5181 | 2HTFA | 7259 |
| 1P9JA | 5801 | 2K5EA | 15833 | 1YSMA | 6498 | 1NY9A | 5706 |
| 2BYEA | 6624 | 2EVNA | 6338 | 2IFSA | 15020 | 2JSXA | 15381 |
| 2BN8A | 5950 | 1INZA | 4959 | 2IKDA | 15105 | 2RNGA | 11022 |
| 2KMGA | 16428 | 1WZ0A | 11267 | 2JV2A | 15465 | 2K87A | 15723 |
| 2KZHA | 17005 | 2L4AA | 17234 | 2LFCA | 17747 | 2KKMA | 16365 |
| 1BV2A | 4917 | 2KE4A | 16129 | 2YU0A | 10275 | 2JTVA | 15419 |
| 2JXTA | 15573 | 2H3KA | 6759 | 2LJ6A | 17283 | 1WGKA | 11258 |
| 1YXEA | 6510 | 2KT8A | 16688 | 1WFIA | 10084 | 2JVEA | 15477 |
| 2JO1A | 16168 | 2K9SA | 16001 | 2EEFA | 10165 | 2KIGA | 16273 |
| 2KVSA | 16794 | 2AFEA | 6751 | 2K0LA | 15651 | 2KS9A | 16660 |
| 1ZXFA | 6726 | 1WJUA | 10050 | 2L8DA | 17402 | 2B1OA | 6159 |
| 2FXPA | 6969 | 1LMZA | 5398 | 2K9KA | 15988 | 2JMUA | 15063 |
| 1IQSA | 5129 | 2AYAA | 6869 | 1SP0A | 6190 | 1WGSA | 10041 |
| 2L25A | 17124 | 2NWTA | 7362 | 1ON4A | 5742 | 1DP3A | 4584 |
| 2G7HA | 7122 | 1KD6A | 4797 | 2ERSA | 6882 | 2KHZA | 16258 |
| 2K5GA | 15835 | 2JQ8A | 15271 | 2KQ5A | 16589 | 1K1ZA | 5179 |
| 1WRGA | 6350 | 2E7CA | 11235 | 2KATA | 16030 | 1QA5A | 4951 |
| 2K3DA | 15750 | 1FJPA | 4907 | 1N6UA | 5049 | 2LBWA | 17578 |

**Table 1.2 Relationship between PDB ID and BMRB ID (Testing Set)**

| **PDB ID** | **BMRB ID** | **PDB ID** | **BMRB ID** | **PDB ID** | **BMRB ID** | **PDB ID** | **BMRB ID** |
| --- | --- | --- | --- | --- | --- | --- | --- |
| 2HJJA | 7261 | 1S62A | 4771 | 2E9GA | 10141 | 2GDTA | 7014 |
| 2JX9A | 15553 | 2H7EA | 7150 | 1PLOA | 4779 | 2LEQA | 17723 |
| 1RSFA | 5516 | 1SS6A | 6189 | 2KGJA | 16211 | 1WH9A | 10095 |
| 2LFPA | 17768 | 1Y4OA | 6396 | 2KCTA | 16096 | 2K5VA | 15849 |
| 2DMMA | 11102 | 2KJQA | 16336 | 1UG7A | 10101 | 2G1EA | 6982 |
| 2KKXA | 16374 | 2KQ1A | 16576 | 1YX6A | 6233 | 1S7AA | 6044 |
| 2PACA | 10132 | 1N3JA | 5567 | 2KP6A | 16545 | 2KCKA | 16083 |
| 2GA5A | 6356 | 2DJKA | 6971 | 1WIEA | 11180 | 1RHXA | 6010 |
| 1WK0A | 10034 | 1Z7RA | 6410 | 1L2MA | 5297 | 2JVFA | 15478 |
| 1XSXA | 5891 | 2K5WA | 15850 | 1WGHA | 11257 | 2KHVA | 16255 |
| 2KPMA | 16560 | 2DJMA | 7083 | 2K3IA | 15762 | 1XKEA | 5159 |
| 2JMHA | 7276 | 1V5MA | 10113 | 2LKYA | 18016 | 1ZDVA | 6629 |
| 1VEEA | 5929 | 1JW3A | 5165 | 2K32A | 15735 | 2GLWA | 7116 |
| 2L50A | 17261 | 2KHAA | 16231 | 2JOVA | 15203 | 2EE0A | 10268 |
| 1IVMA | 4751 | 2ABYA | 6755 | 2K5DA | 15829 | 1EOQA | 4593 |
| 2KCAA | 16068 | 1G9LA | 4915 | 2K3UA | 15778 | 2KVQE | 16788 |
| 2KLAA | 16389 | 2D9WA | 10279 | 2OT2A | 15152 | 2KYHA | 16957 |
| 1FHOA | 4373 | 2HC5A | 7170 | 1Z8SA | 6716 | 3MSPA | 4242 |
| 2BO5A | 6564 | 2JRHA | 15332 | 1IIOA | 4996 | 2L8OA | 17420 |
| 2GMOA | 7056 | 2L48A | 17233 | 1T17A | 6120 | 2LE0A | 17687 |
| 2KHQA | 16251 | 2L7UA | 17378 | 1OVQA | 5758 | 1WGWA | 10043 |
| 1BCIA | 4188 | 2KL6A | 16385 | 1WH4A | 10092 | 2F09A | 6955 |
| 2I4KA | 7302 | 2L76A | 17345 | 2L1TA | 17105 | 1RZWA | 6058 |
| 1RMJA | 5545 | 2KLQA | 16396 | 1Y9OA | 6398 | 2K8YA | 15981 |
| 2K0DX | 15645 | 2JOZA | 15211 | 2K5JA | 15839 | 2V31A | 7053 |
| 2KY9A | 16942 | 2KZ3A | 16996 | 2L9QA | 17482 | 2JODA | 15166 |
| 1XHJA | 6355 | 2LF0A | 17735 | 2O3DA | 7301 | 1G9EA | 4628 |
| 1T4ZA | 5141 | 2KD1A | 16102 | 1L7YA | 5329 | 2KXGA | 16913 |
| 2L1PA | 17092 | 1LL8A | 5354 | 2K02A | 15634 | 2KD2A | 16103 |
| 2JQNA | 15281 | 1TTNA | 6609 | 1LKNA | 5357 | 2L6EA | 17307 |
| 2JOYA | 15210 | 1WFMA | 10074 | 2KXQA | 16923 | 2L05A | 17030 |
| 1JNJA | 5169 | 2KFSA | 16188 | 2RQKA | 11076 | 2K8QA | 15958 |
| 1YR1A | 6395 | 2DHZA | 11277 | 2LEZA | 17734 | 1M3GA | 5552 |
| 2KRKA | 16640 | 1V32A | 10106 | 2K4ZA | 15816 | 2KQ2A | 16578 |
| 1IK0A | 5004 | 2K3JA | 15763 | 2LFRA | 17775 | 2JW1A | 15504 |
| 2KP7A | 16549 | 2AQFA | 6781 | 2F1EA | 5998 | 1KATV | 5185 |
| 2KCOA | 16089 | 2OA4A | 7371 | 2JOBA | 15622 | 1TVMA | 6259 |
| 2DA6A | 10288 | 2KW2A | 16805 | 1L3GA | 4254 | 2V6ZM | 7419 |
| 2JUOA | 15451 | 1UB1A | 4467 | 2KPTA | 16569 | 2K3GA | 15956 |
| 1PA4A | 5796 | 1D8JA | 4721 | 1WHNA | 10125 | 1WJRA | 10031 |
| 1VD0A | 5807 | 1BTAA | 5293 | 1SLJA | 6122 | 2JN7A | 15088 |
| 2L8YA | 17431 | 2KKZA | 16376 | 2K6QA | 15877 | 2L5QA | 17280 |
| 2GW6A | 6860 | 2KK0A | 16348 | 1JRMA | 5104 | 1KA6A | 5212 |
| 2KMTA | 16135 | 2KJGA | 16320 | 1SJQA | 6178 | 1SE7A | 6127 |
| 1BJXA | 4156 | 1BQ0A | 4228 | 2JNAA | 15090 | 1X3BA | 11311 |
| 2JS1A | 15350 | 1MP1A | 5162 | 1WJTA | 10049 | 2JUAA | 15437 |
| 1EHXA | 4589 | 2L9SB | 17485 | 1N3GA | 5389 | 1T0GA | 6138 |
| 2AN7A | 4792 | 2KHMA | 16249 | 2DZKA | 11281 | 2BGOA | 6475 |
| 2A2PA | 6739 | 1SB0A | 6095 | 2FFTA | 6926 | 2L08A | 17033 |
| 2KPPA | 16563 | 2JN6A | 15086 | 2LJWA | 17965 | 2KO6A | 16490 |
| 2L9WA | 17490 | 1F7WA | 4717 | 2KQRA | 16610 |  |  |
