# Supplementary material for: NMRDSP: An Accurate Prediction of Protein Shape Strings from NMR Chemical Shifts and Sequence Data
Source: PLoS One. 2013 Dec 23;8(12):e83532. doi: 10.1371/journal.pone.0083532 (PMC3871590; doi:10.1371/journal.pone.0083532)
Supplement: Supplementary Materials S2 — DSP web server. (DOC) [file pone.0083532.s002.doc]

**Supplementary Materials**

**S2 DSP web server**

DSP [1] is an accurate predictor of protein shape string based on two innovative technologies: a knowledge-driven sequence alignment and a sequence shape string profile method.


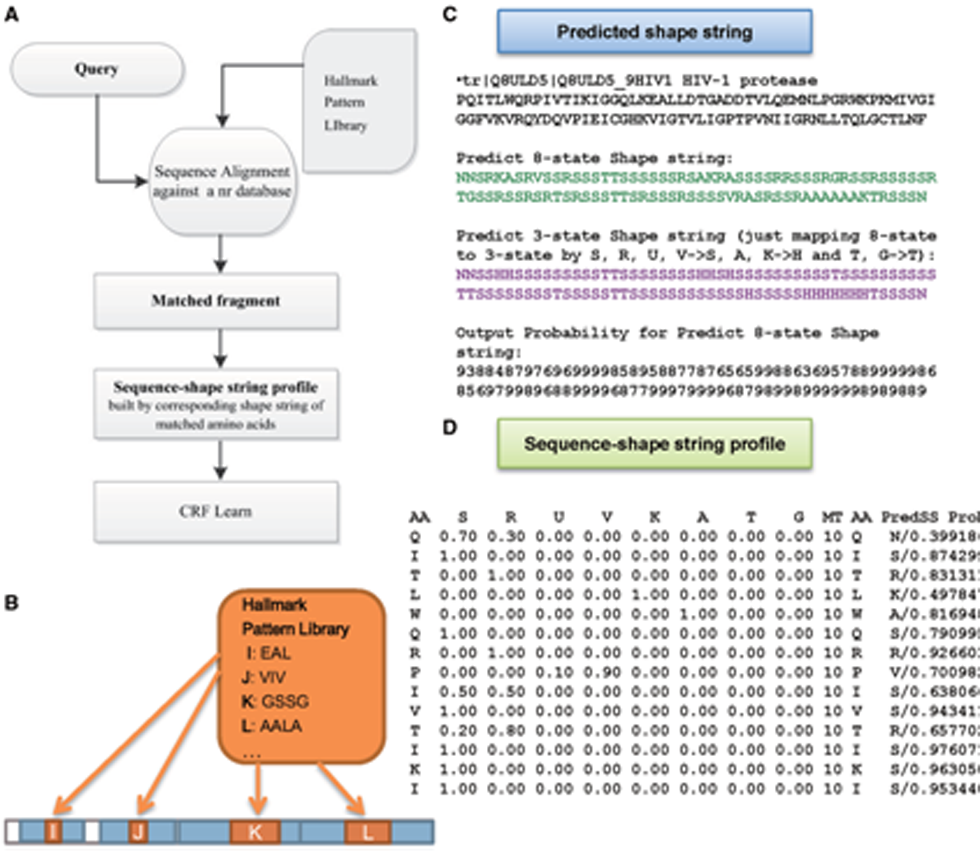


**Figure S2.1. (A)The flowchart of the prediction of shape string and (B) sequence alignment with hallmark patterns as seeds. An example of (C) the predicted shape string and (D) the output sequence shape string profile. AA: amino acid; MT, match times; PredSS, predicted shape string; Prob, output probability.**

The flowchart of the shape string prediction is shown in Figure S2.1 A. The PSI-BLAST algorithm was initially employed to match a query sequence against a protein database constructed by a non-redundant PDB chain set (nr3PDB, NCBI MMDB 2009 December, 40,849 entries in total), resulting in two parts: matched fragments and unmatched fragments. Then, we utilized the hallmark patterns in the hallmark pattern library (HPL) (see below) to hit the unmatched fragments and obtain the hit segments (Figure S2.1 B). These hit segments and their flanking amino acids (+n and -n, default is 5 in this study) were aligned together against nr3PDB using PHI-BLAST, which found more matched shorter sequences. The matched fragments obtained by the first alignment and the shorter sequences obtained by the subsequent alignments were encoded based on corresponding shape string element profiles. The shape string element profile was composed of eight elements, which was employed as feature for predicting the shape string of the query. Lastly, conditional random field (CRF) was performed for modeling and prediction.

**Knowledge-driven sequence alignment**

One innovative character of our approach was a knowledge-driven sequence alignment guided by seeds in a constructed HPL, which was instrumental in searching structural similarities among highly divergent proteins. Initially, we began a traversal search for consecutive sequence patterns with sufficient frequency in a representative non-redundant PDB chain set (nr0PDB, NCBI MMDB 2009 Dec, 7775 entries, 0-level non-redundancy, two sequences are considered similar if they have a BLAST E-value of 10^-7^ or less). In our previous study [2], we introduced an algorithm that could extract local combinational variables with fixed locations from equal length sequences. Here, the algorithm was developed to extract candidate patterns from unequal length sequences without sequence alignment. These short patterns were merged with every other single fragment that contained the same residue as the former fragment in order to form potentially longer sequences while maintaining the frequency criterion. We set the frequency criterion to 100 and a total of 5667 consecutive sequence patterns were obtained. The entire pattern extraction process progressed as the fragment grew longer, a process known as the bottom-up method.

Second, hallmark patterns were defined as conservative both in sequence patterns and shape string structures. For each position of a consecutive sequence pattern, the P-value of the corresponding shape string of the amino acid at this position was calculated according to a binomially distributed model.

Thirdly, based on the P-values, we selected 2761 hallmark patterns with lengths ranging between 2 and 4 residues that typically exhibited conserved structures to construct the library. The HPL represented remote homology in the sequences and shape strings and was an indispensable tool in our approach.

**Sequence shape string profile**

The sequence shape string profile was another innovative character of our approach, which was generated as follows: In the first step, the query sequence was aligned against the nr3PDB (NCBI MMDB 2009 December, 3-level non-redundancy, 40 849 entries in total) resulting in the top n (n=10 in this work) subjects. Then, the shape strings of the n subjects were retrieved. Finally, the shape string elements of every amino acid were counted and stored in eight boxes (Figure S2.1 D). These boxes constituted a vector that represents the sequence shape string profile for each residue and was considered to include the structural evolutionary information more details about sequence structure profile can be found in our previous study [3].

**References:**

1. Sun, J., et al., *DSP: a protein shape string and its profile prediction server.* Nucleic Acids Res, 2012. **40**(Web Server issue): p. W298-302.

2. Xiong, W., et al., *Local combinational variables: an approach used in DNA-binding helix-turn-helix motif prediction with sequence information.* Nucleic Acids Res, 2009. **37**(17): p. 5632-40.

3. Li, D., et al., *A novel structural position-specific scoring matrix for the prediction of protein secondary structures.* Bioinformatics, 2012. **28**(1): p. 32-9.
