# Supplementary material for: NMRDSP: An Accurate Prediction of Protein Shape Strings from NMR Chemical Shifts and Sequence Data
Source: PLoS One. 2013 Dec 23;8(12):e83532. doi: 10.1371/journal.pone.0083532 (PMC3871590; doi:10.1371/journal.pone.0083532)
Supplement: Supplementary Materials S3 — Structural Position-Specific Scoring Matrix. (DOC) [file pone.0083532.s003.doc]

**Supplementary Materials**

**S3 Structural Position-Specific Scoring Matrix (SPSSM)**

SPSSM was proposed for prediction of protein secondary structure [1]. SPSSM is a distinctive PSSM-like profile which contains evolutionary information of protein secondary structure. The generation of SPSSM is described in Figure S3.1.


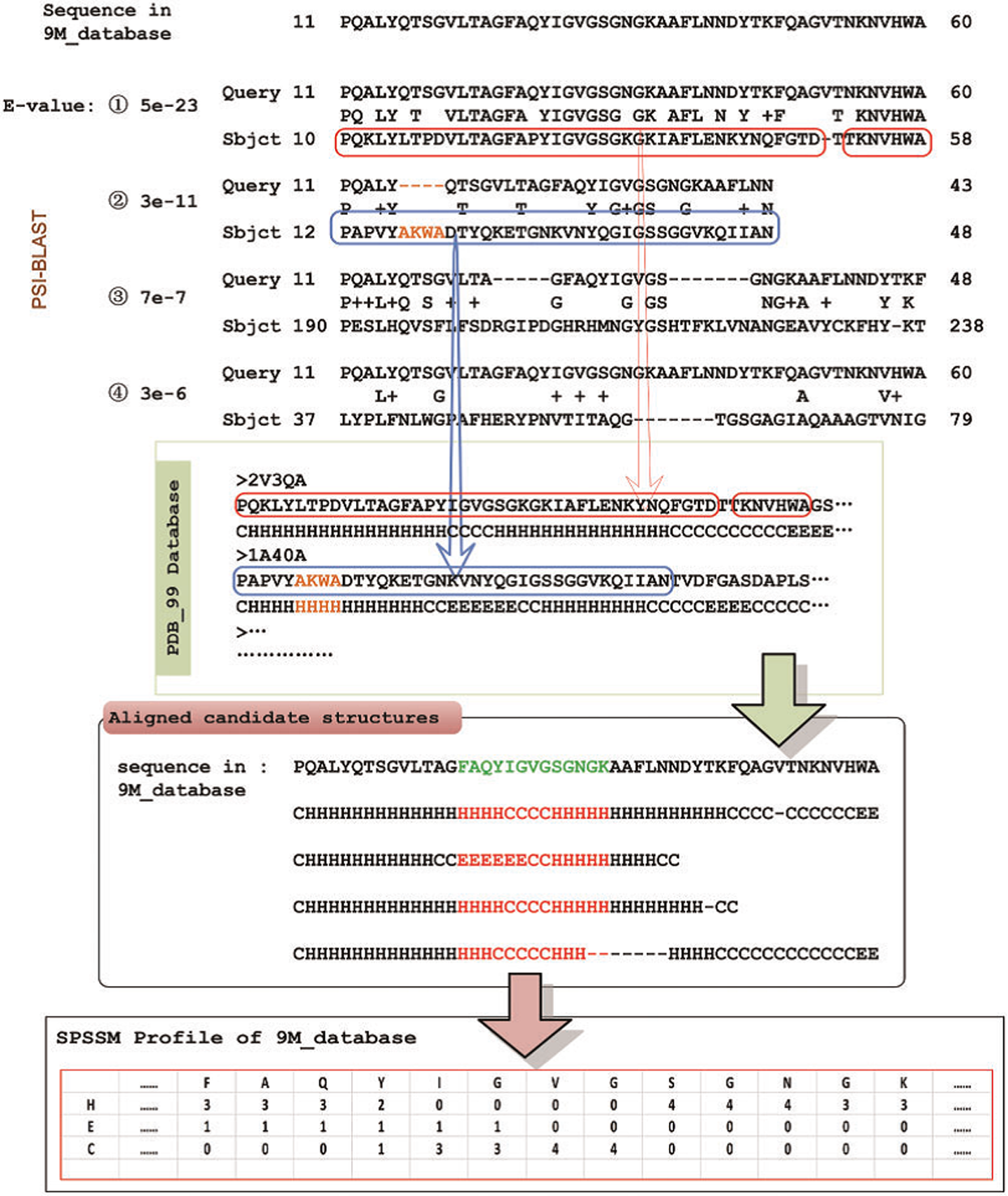


**Figure S3.1 The generation of SPSSM.**

Before generating SPSSM, 9M_database was constructed. The 9M_database is a BLAST compatible database in which there are 9 million sequences and corresponding secondary structural profiles. The sequences in the 9M_database were derived from the non-redundant NCBI database (as of 2009, 9 069 431 proteins) applied in PSIBLAST. The secondary structural profiles of the 9M_database were generated by alignment and score. Each of the 9 million sequences was aligned against a non-redundant PDB (70,177 proteins) with PSI-BLAST by setting the *e*-value at 1e-5 and other parameters at default. The secondary structure elements in the matched unions were scored in three boxes that contained the scores of three state secondary structural elements. These boxes then constituted a secondary structural profile of the original sequence. This procedure was repeated until the profiles of all sequences in the 9M_database were formed.

For a query PSI-BLAST is carried out firstly (Figure S3.1 up) to align against 9M_database. The secondary structure profiles of the matched sequences are collected (Figure S3.1 middle). Then, for each amino acid in the query sequence the score is calculated as the sun of all obtained profiles (Figure S3.1. bottom). This is raw SPSSM. When SPSSMPred [1] is carried out the output is normalized and one will obtain normalized SPSSM.

The SPSSM reflects the sequence alignment’s shapes, and the properties of the secondary structure. The SPSSM is the expansion of the PSSM with regard to the structural aspects; the SPSSM inherits the concepts of the PSSM, but takes more consideration of the deeper common ground beneath the aligned sequences, where structural information may provide extra clues for regularity.

SPSSM has been confirmed as an effective feature for prediction of protein structure and function. In predicting turns in proteins with a unified model [2] SPSSM was used as a feature.

**References:**

1. Li D, Li T, Cong P, Xiong W, Sun J (2012) A novel structural position-specific scoring matrix for the prediction of protein secondary structures. Bioinformatics 28: 32-39.

2. Song Q, Li T, Cong P, Sun J, Li D, et al. (2012) Predicting turns in proteins with a unified model. PLoS One 7: e48389.
