# Supplementary figures and images for: NMRDSP: An Accurate Prediction of Protein Shape Strings from NMR Chemical Shifts and Sequence Data
Source: PLoS One. 2013 Dec 23;8(12):e83532. doi: 10.1371/journal.pone.0083532 (PMC3871590; doi:10.1371/journal.pone.0083532)

**Supplementary Materials**

**S4 The distributions of NMR after normalization for residues**


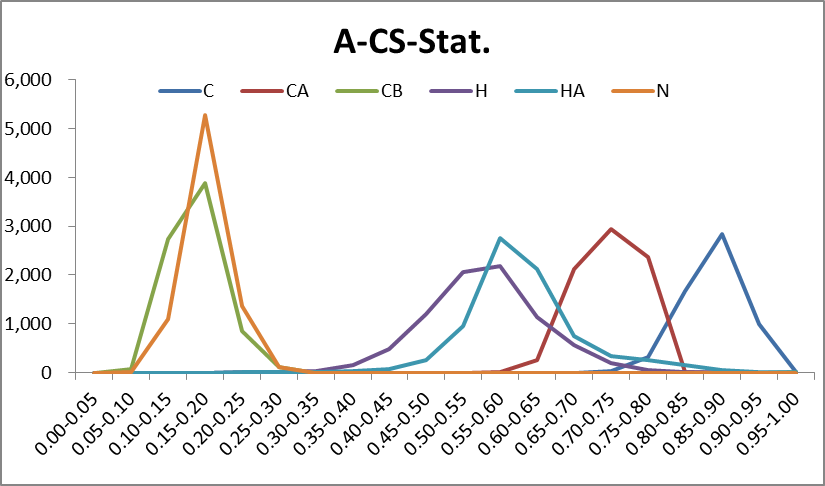


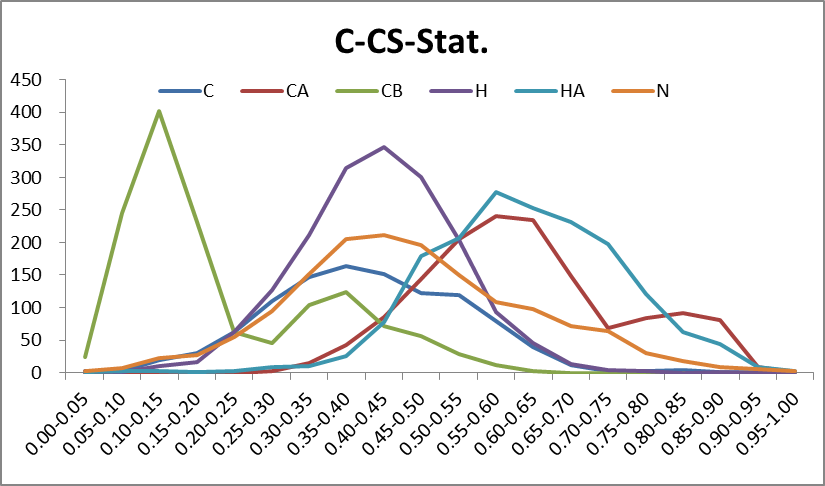


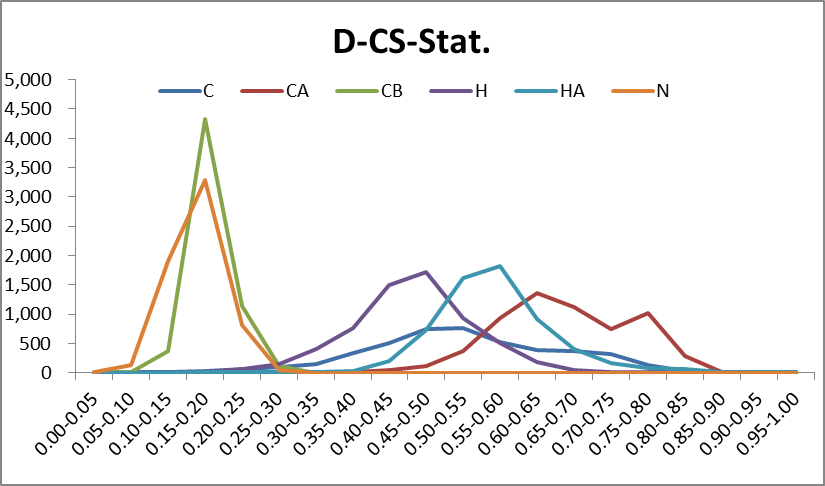


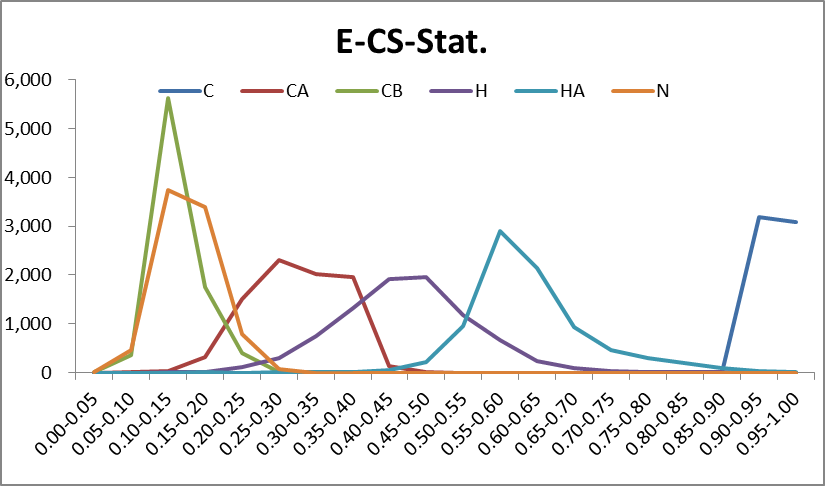


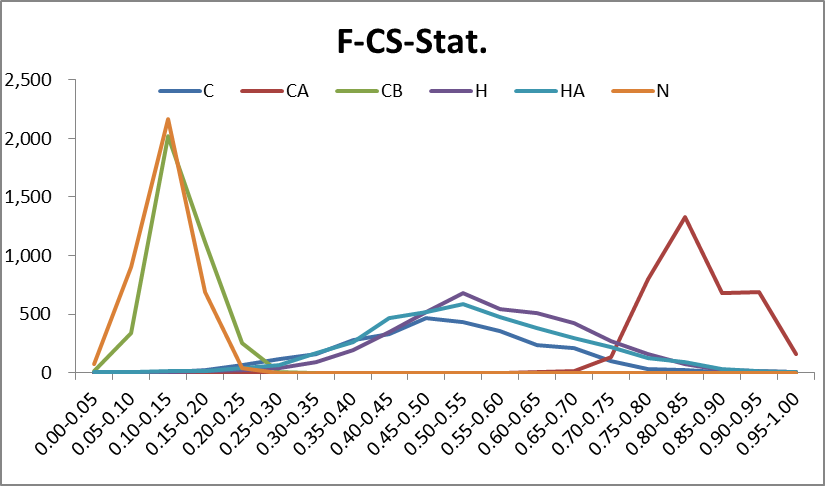


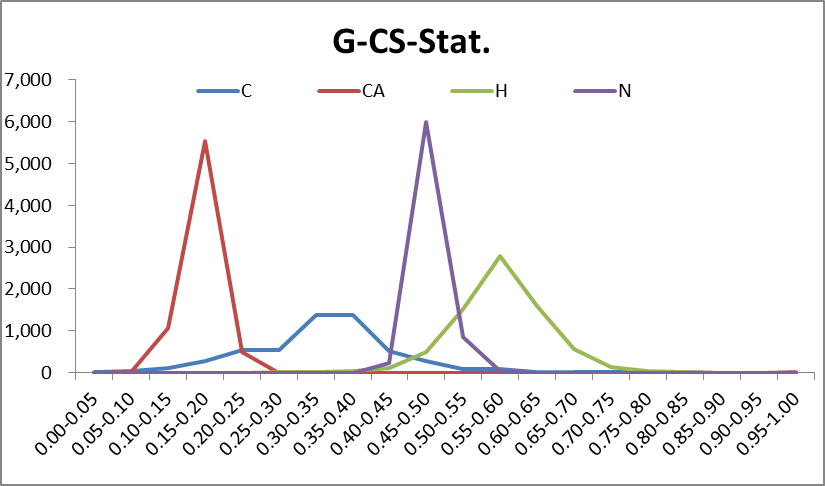


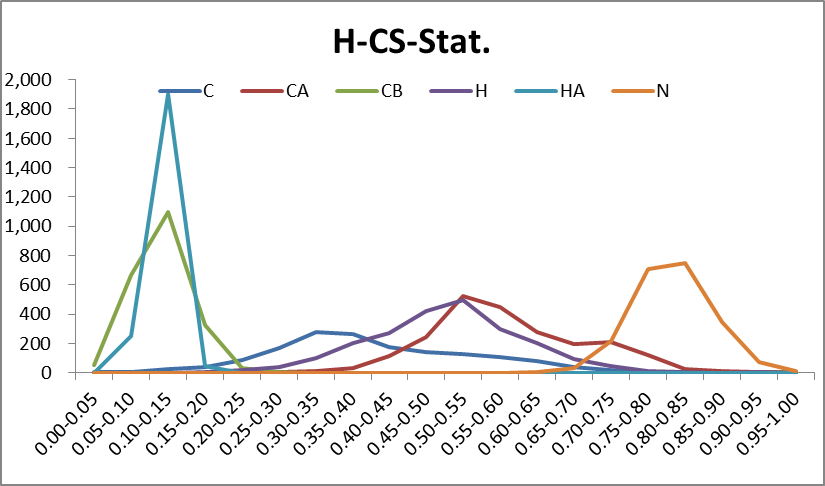


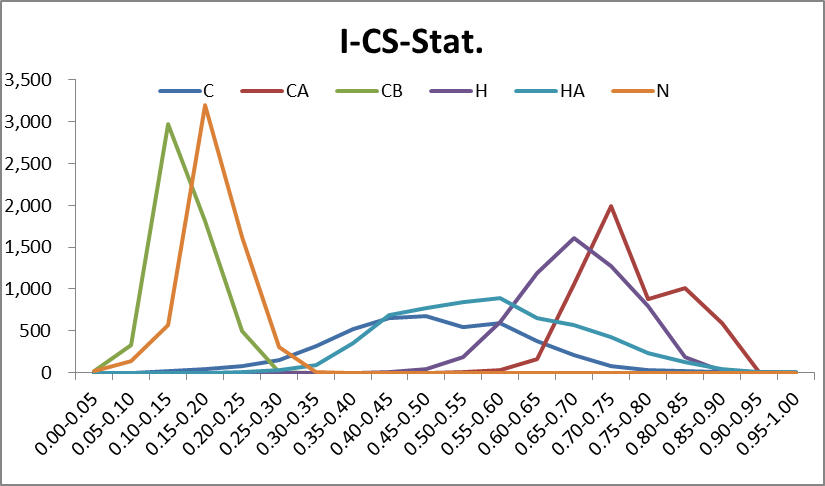


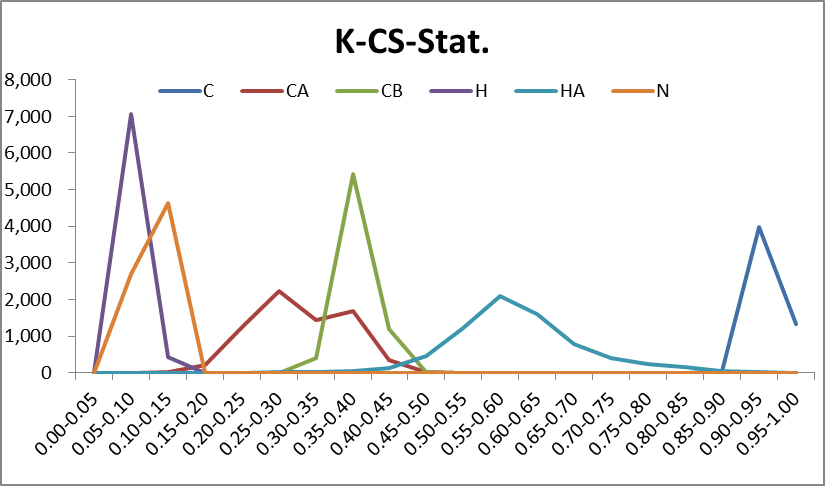


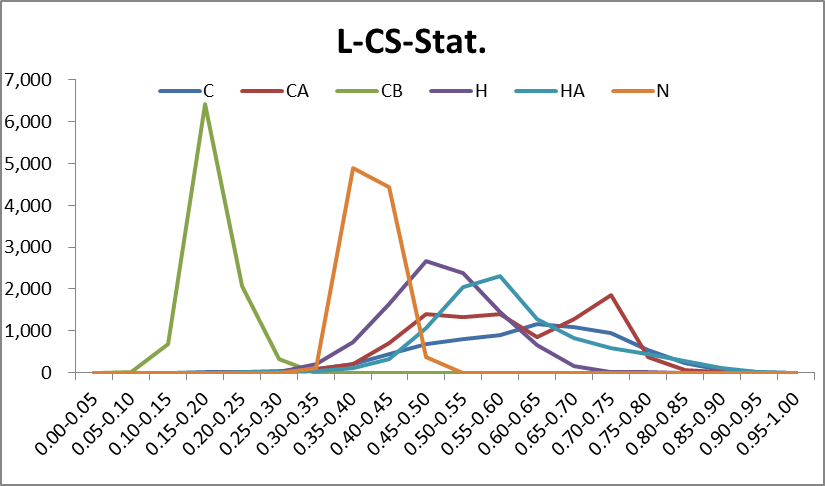


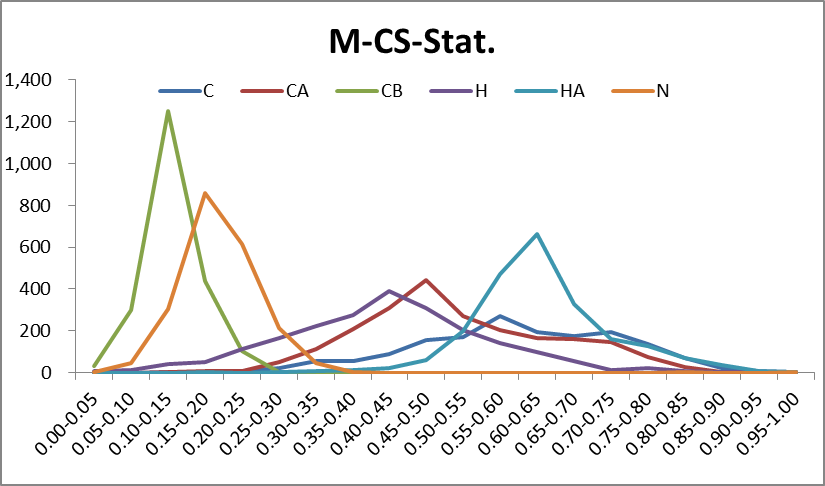


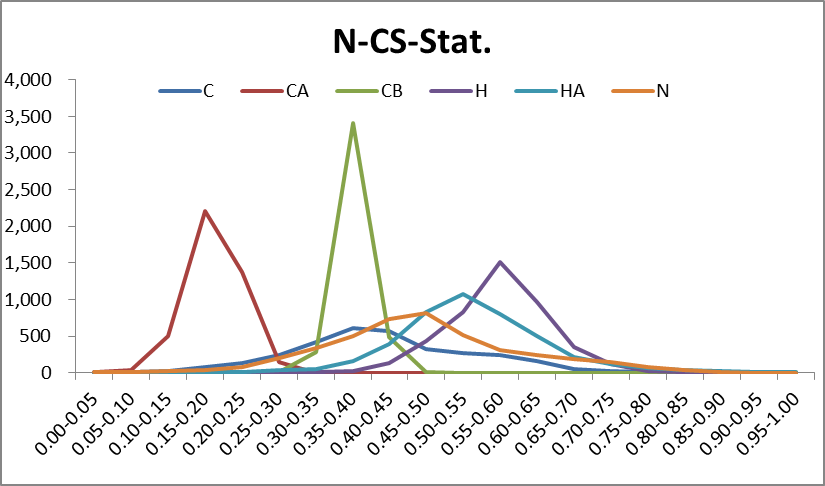


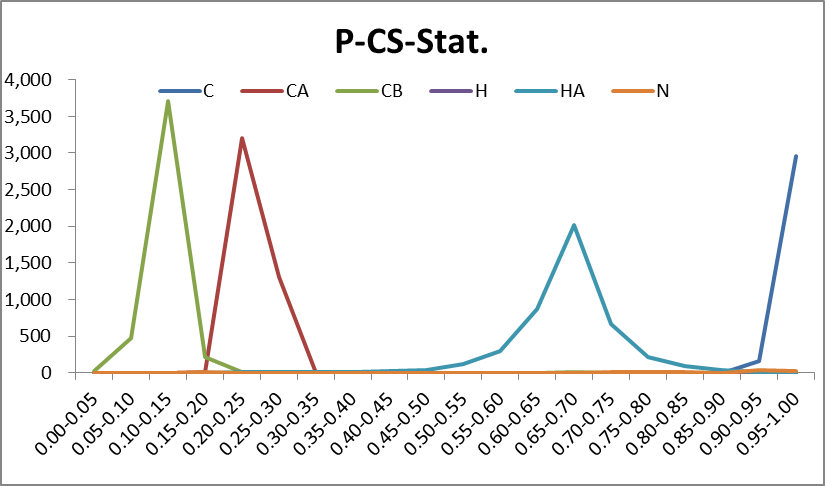


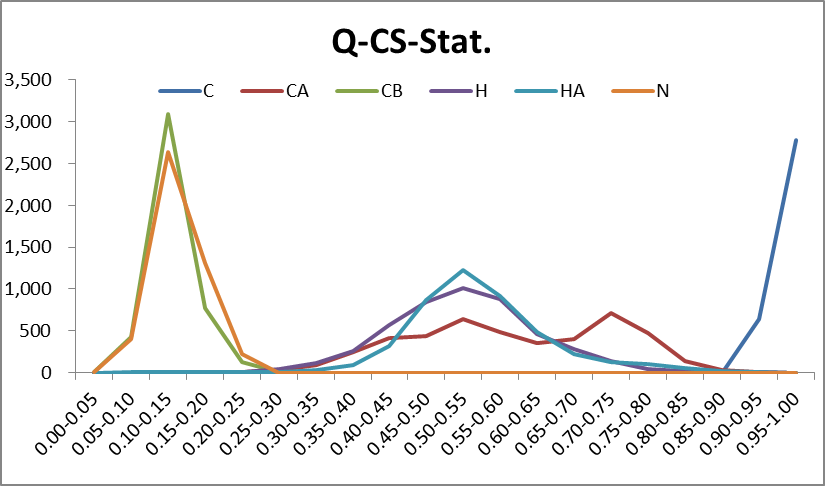


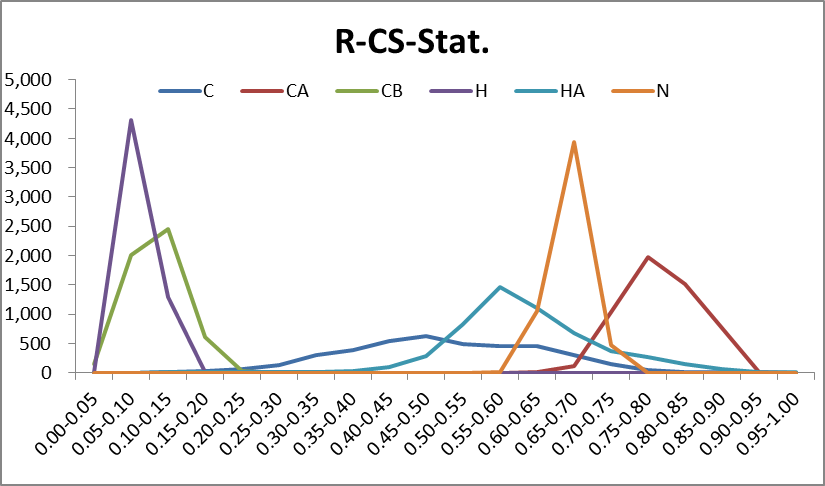


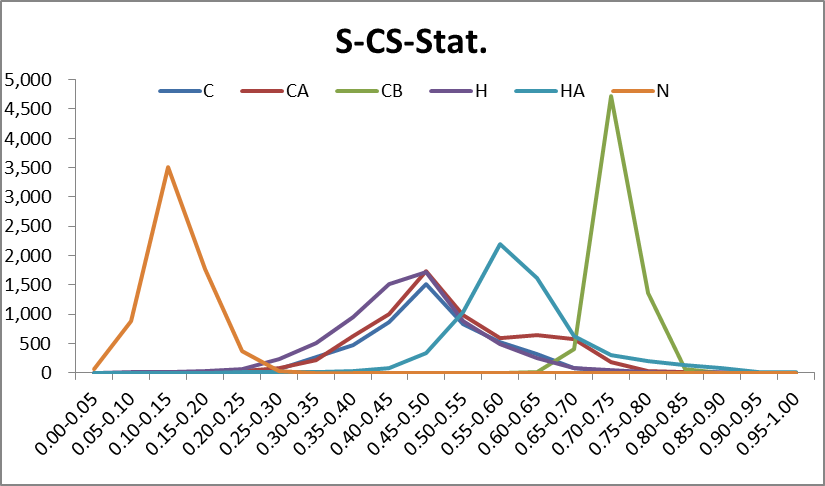


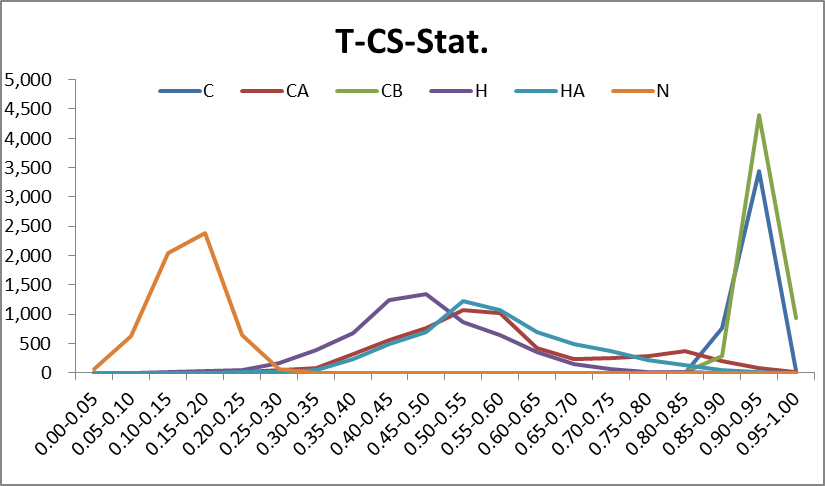


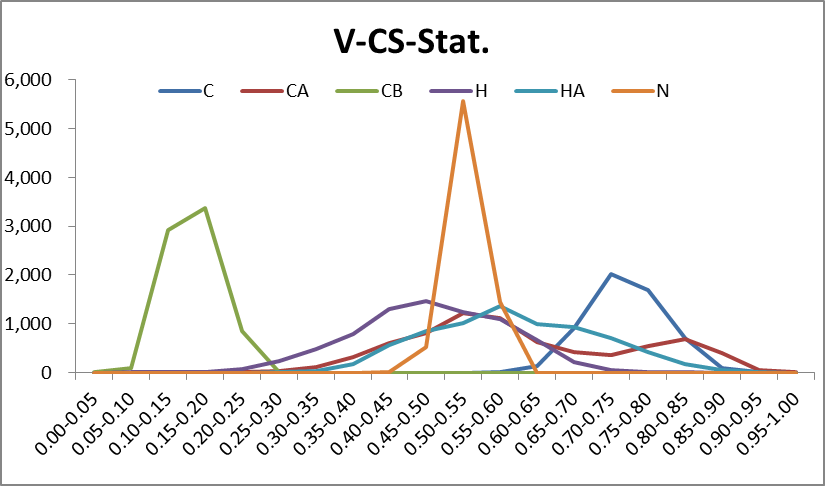


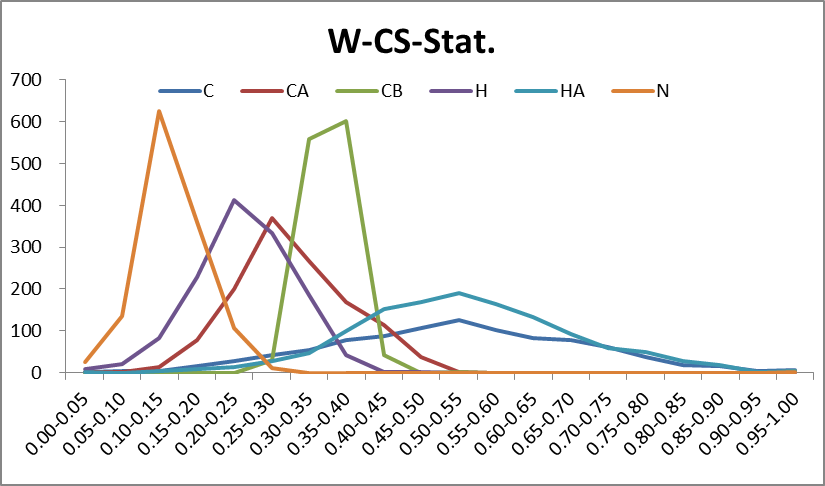


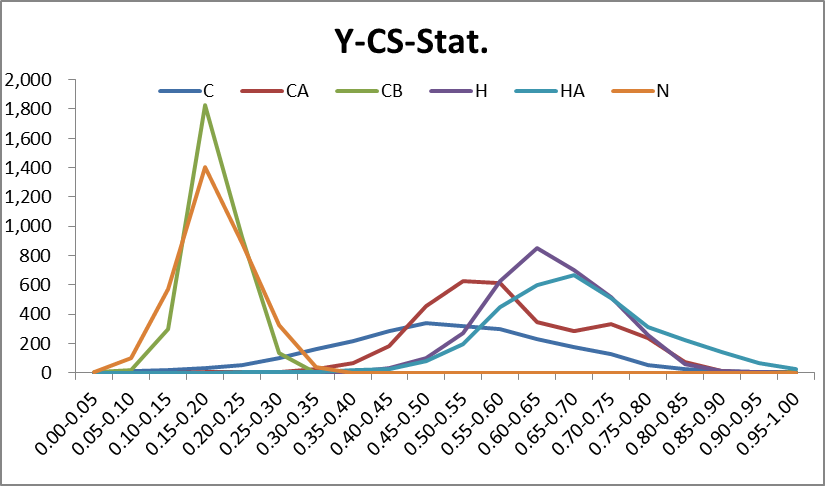

Supplement: Supplementary Materials S4 — The distributions of NMR after normalization for residues. (DOC) [file pone.0083532.s004.doc]
