# Supplementary material for: NMRDSP: An Accurate Prediction of Protein Shape Strings from NMR Chemical Shifts and Sequence Data
Source: PLoS One. 2013 Dec 23;8(12):e83532. doi: 10.1371/journal.pone.0083532 (PMC3871590; doi:10.1371/journal.pone.0083532)

**Supplementary Materials**

**S5 The distributions of NMR CS data after normalization for shape strings**

## HA


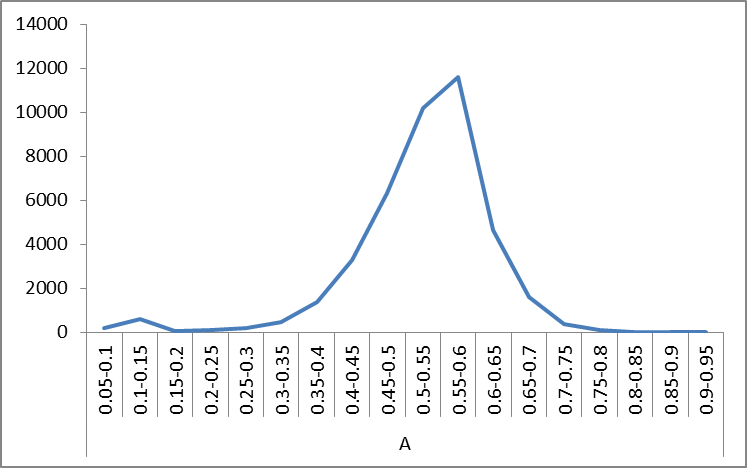


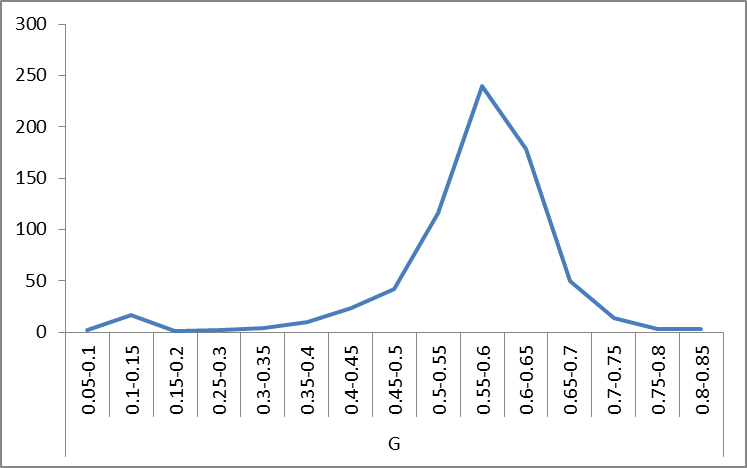


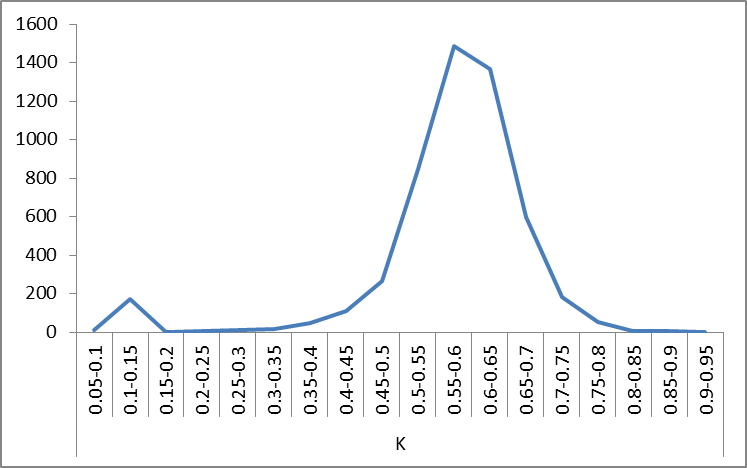


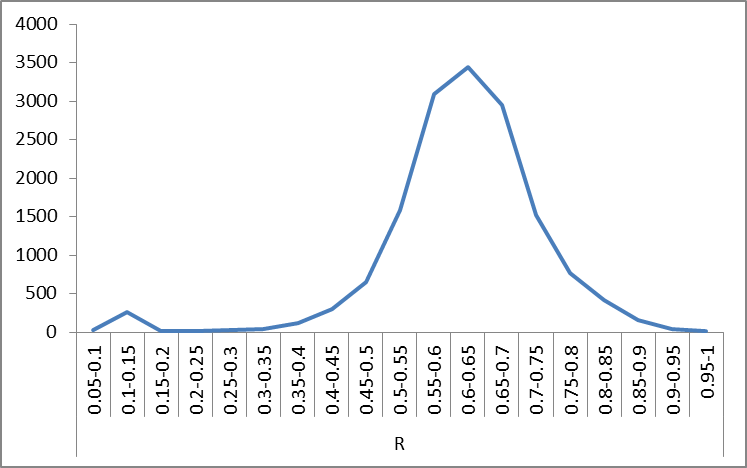


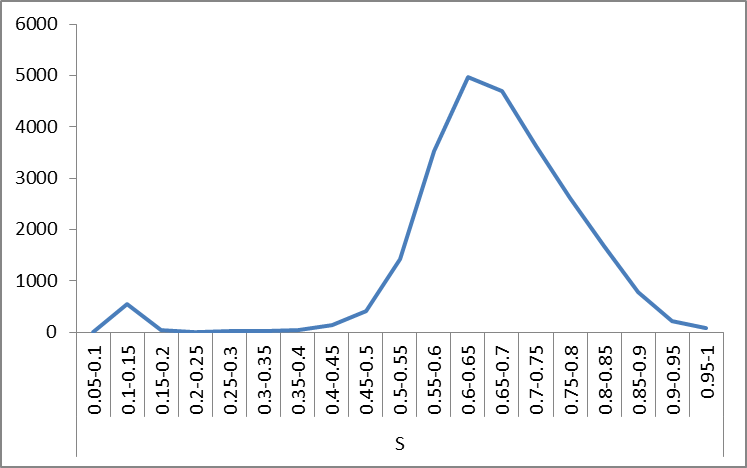


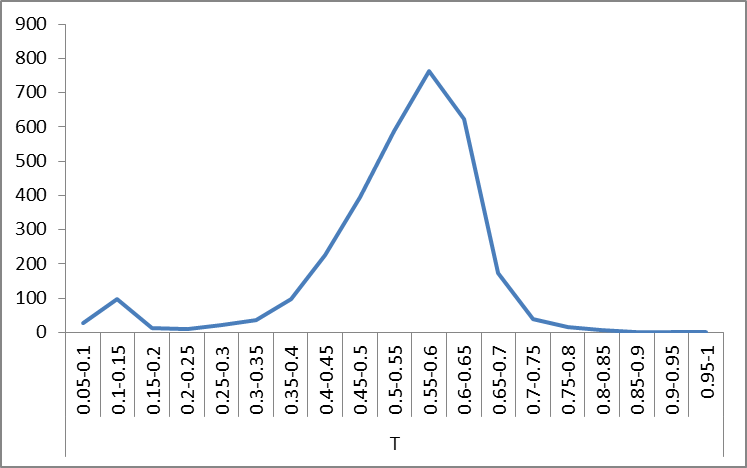


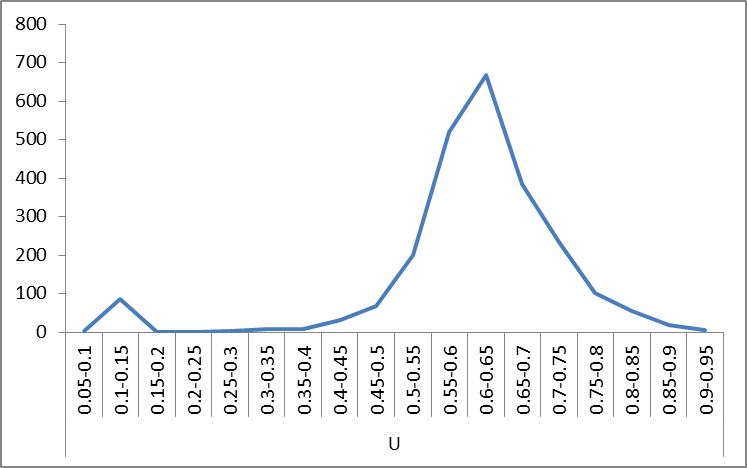


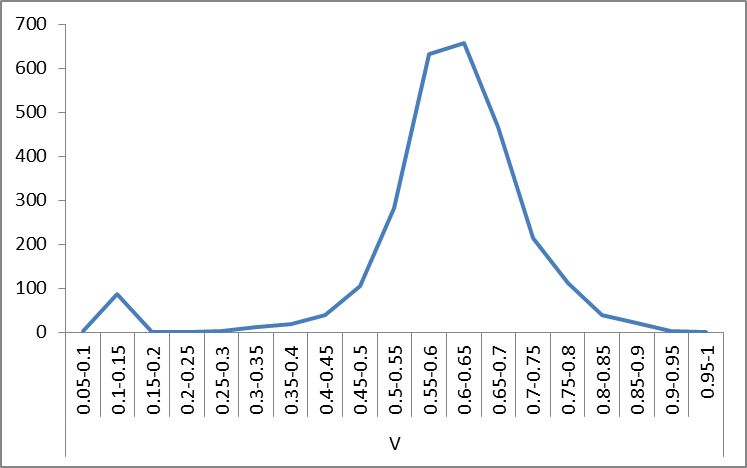


## H


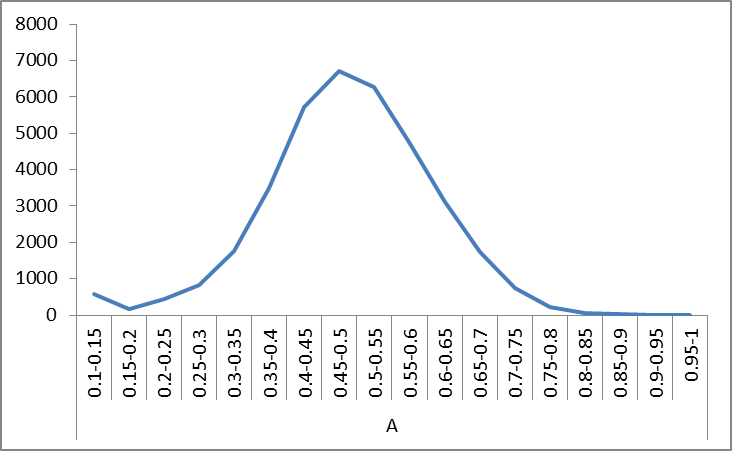


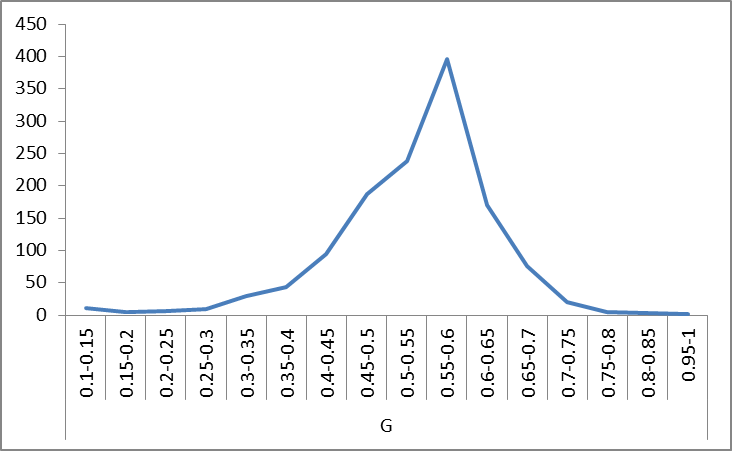


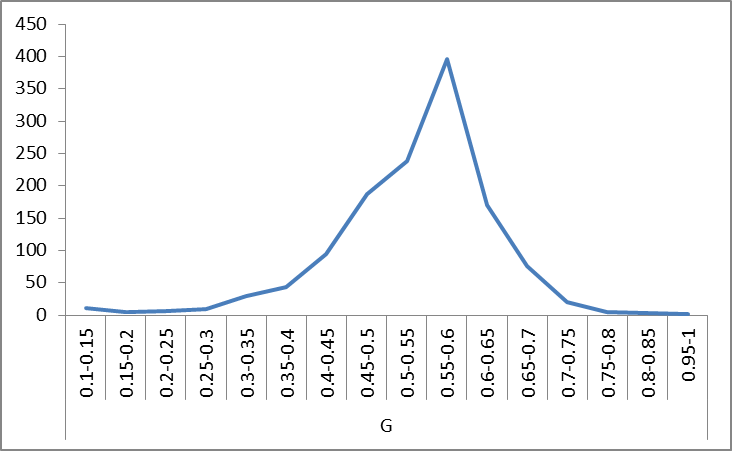


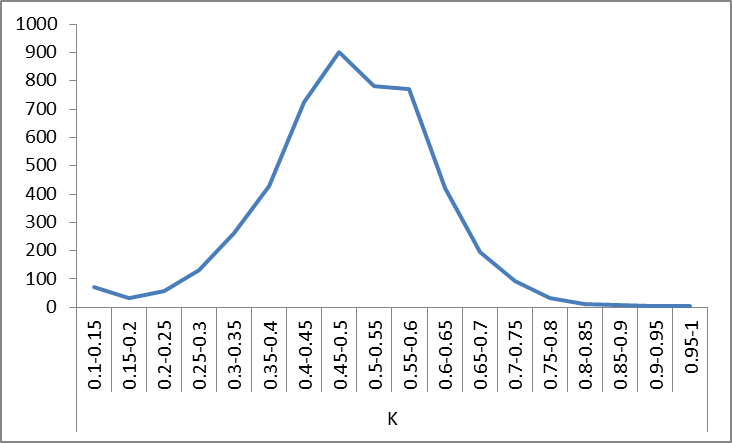


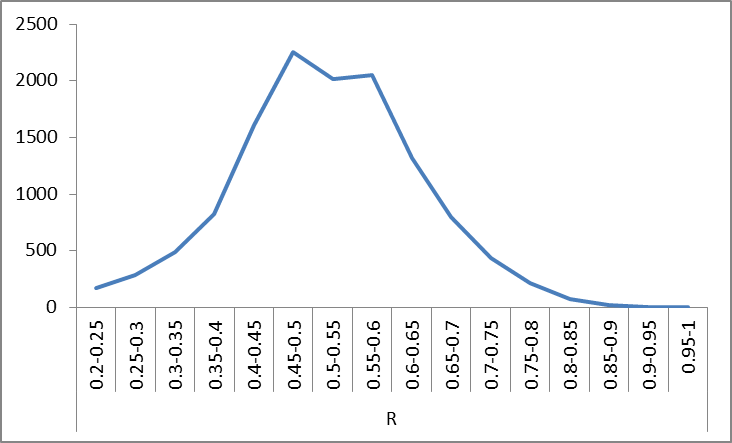


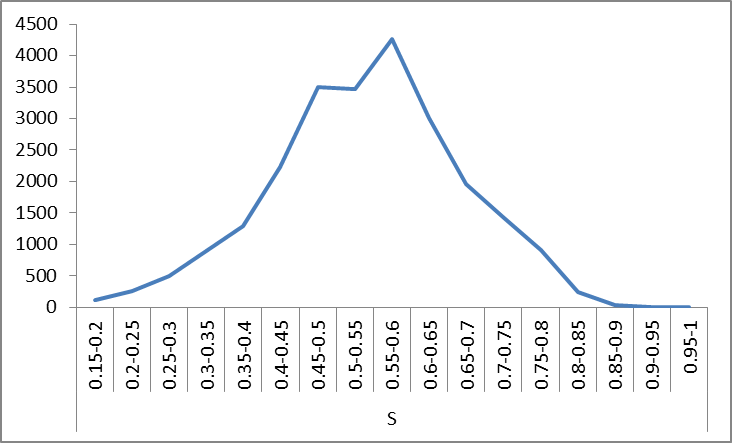


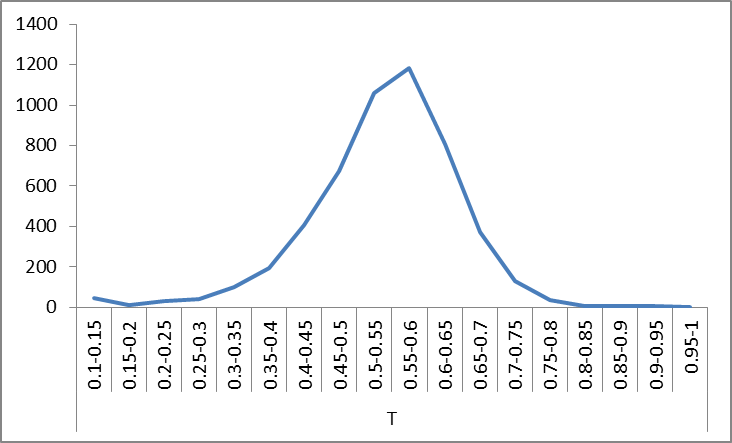


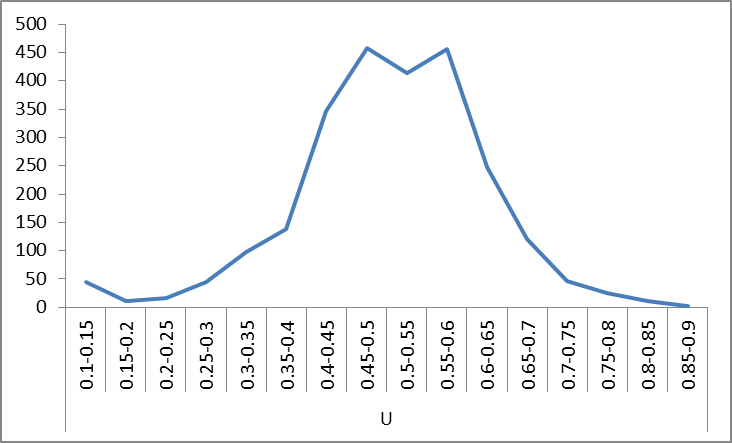


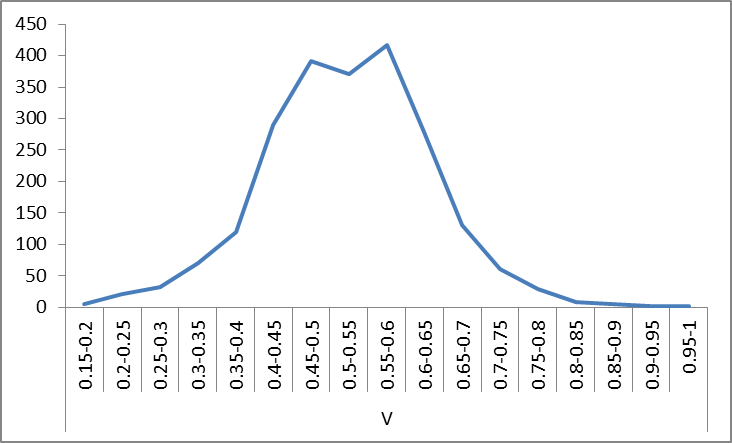


## N


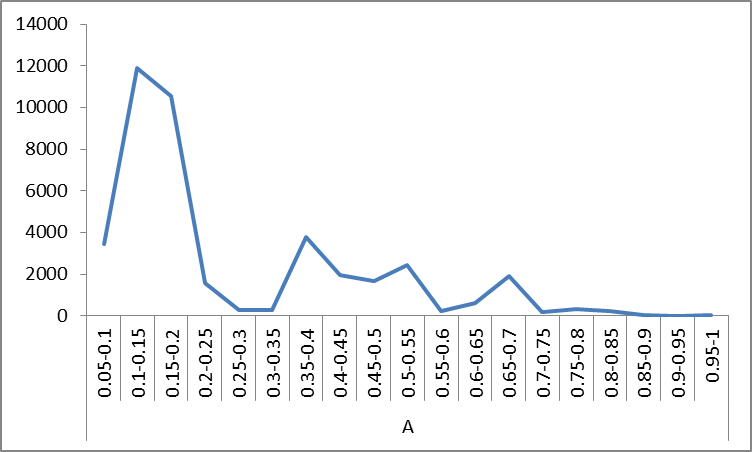


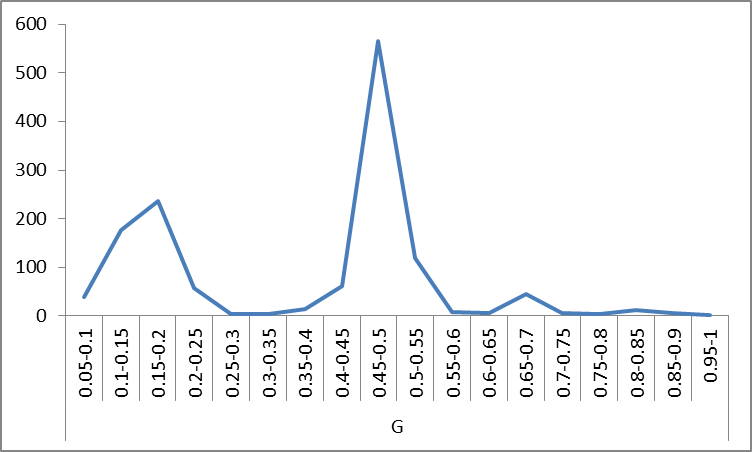


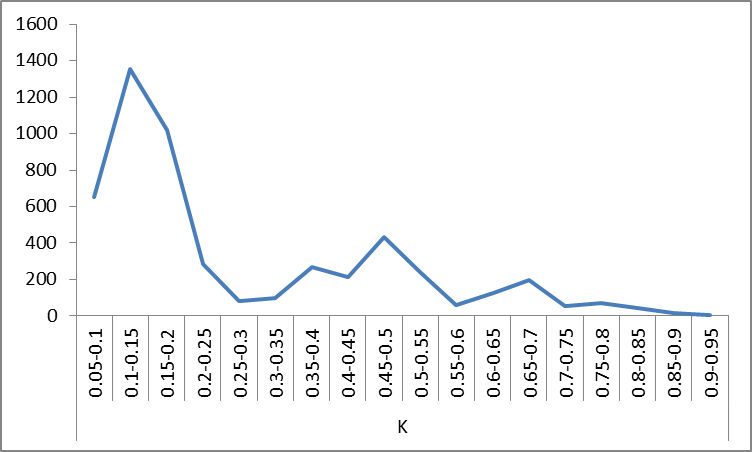


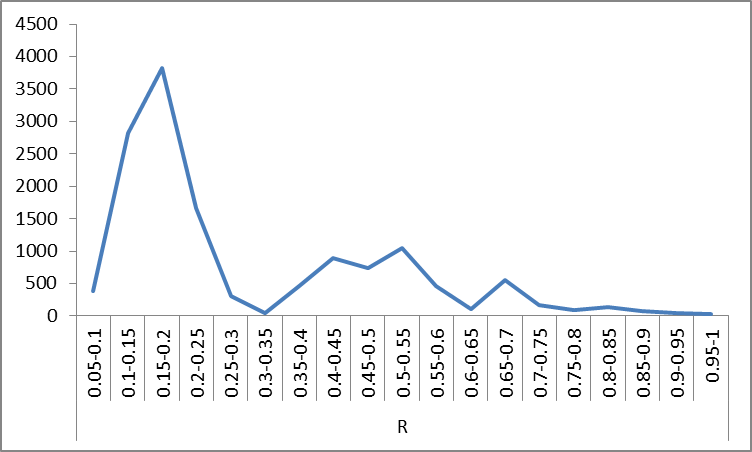


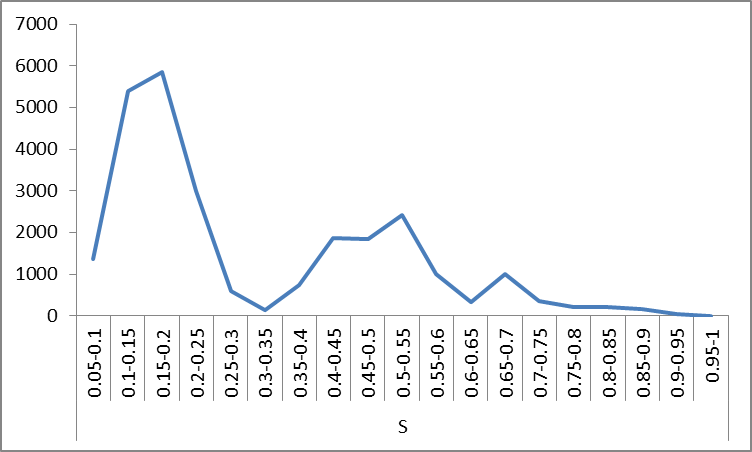


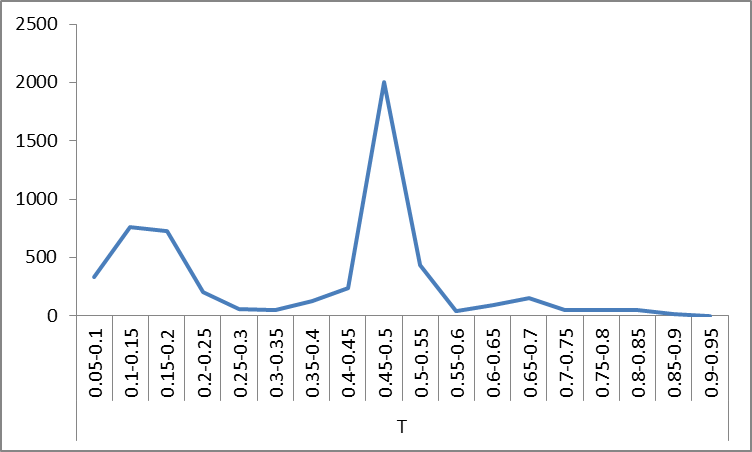


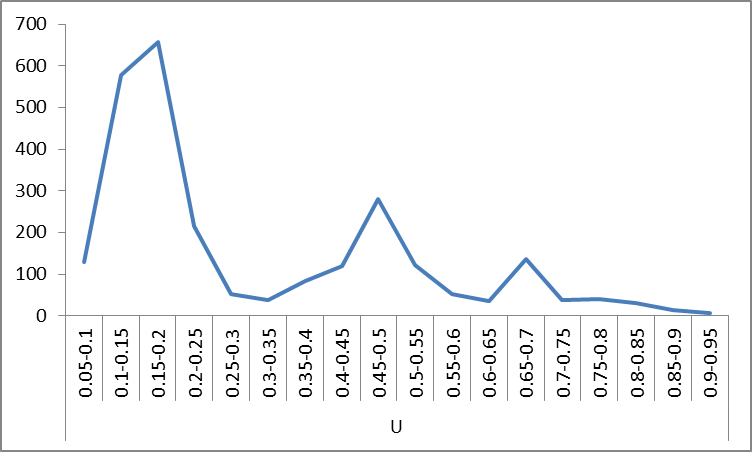


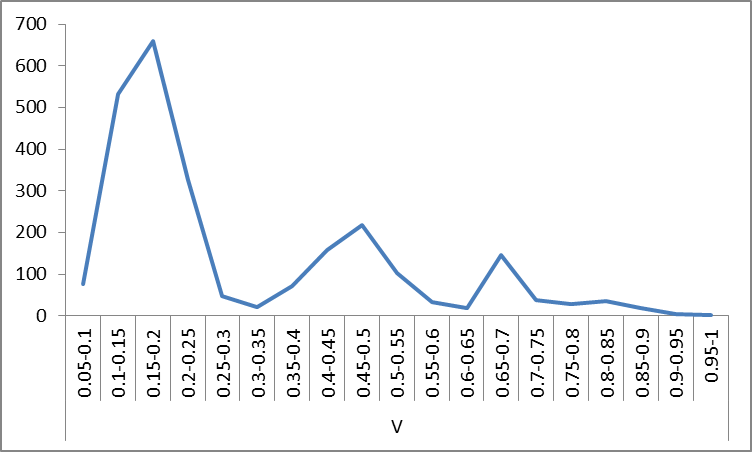


## CA


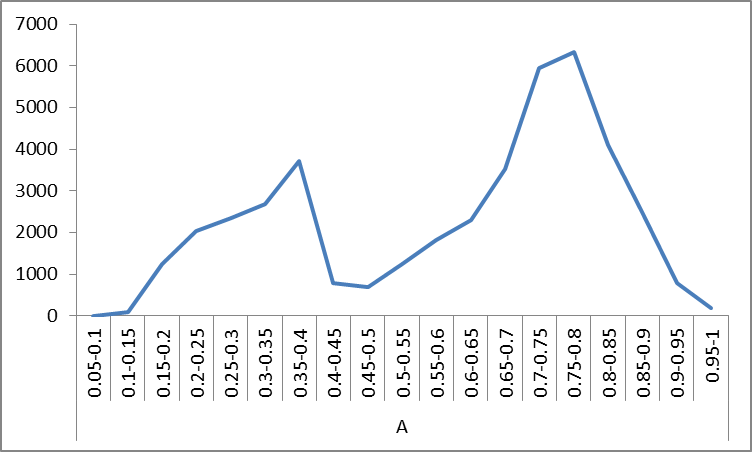


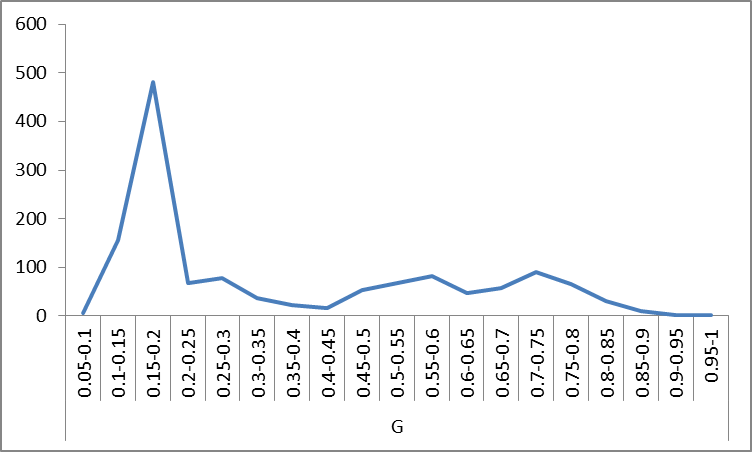


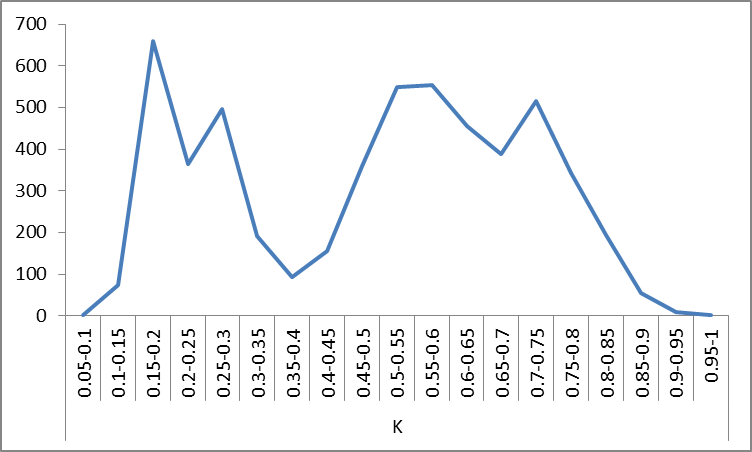


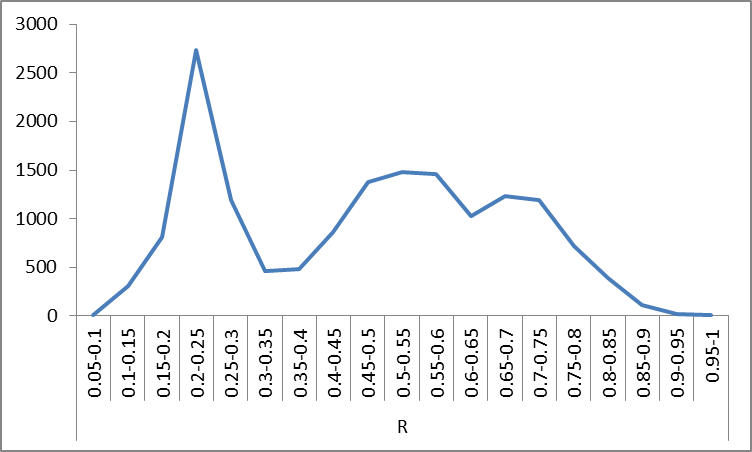


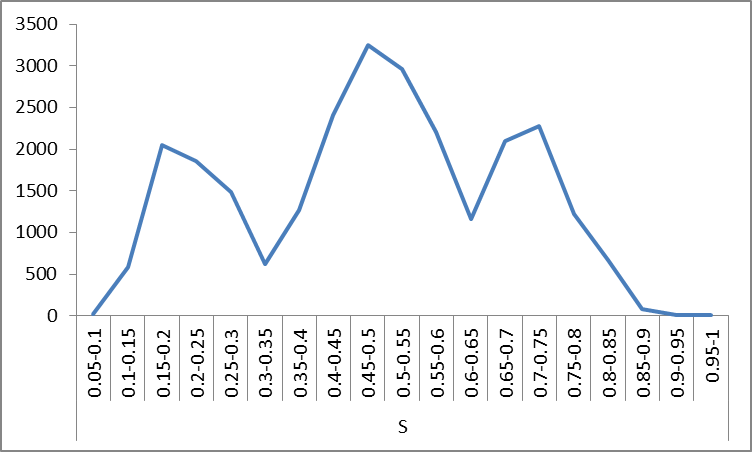


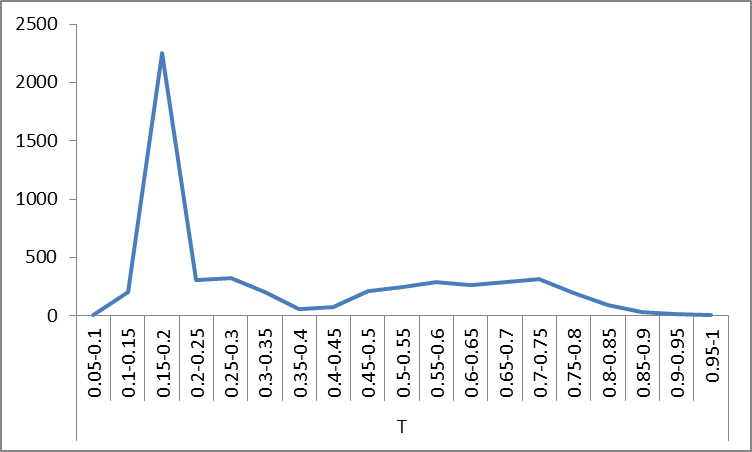


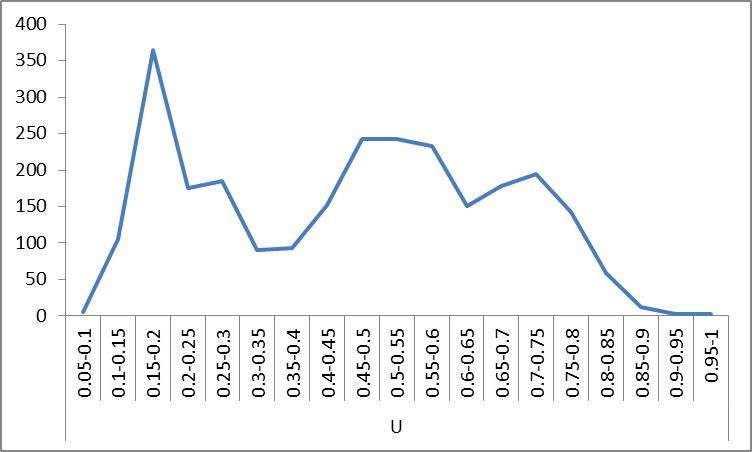


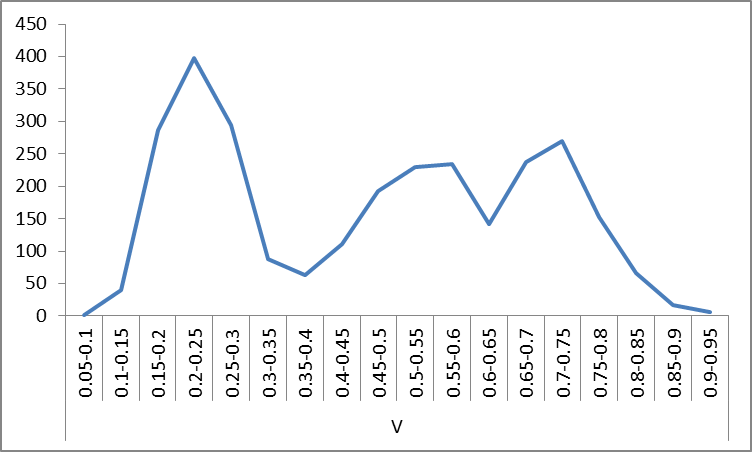


## CB


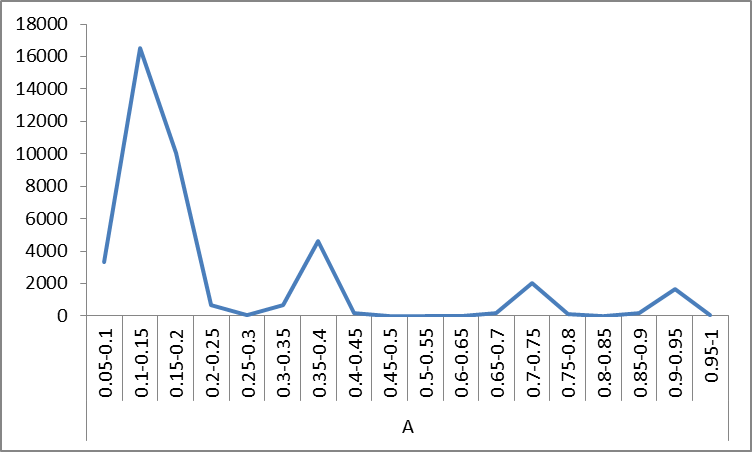


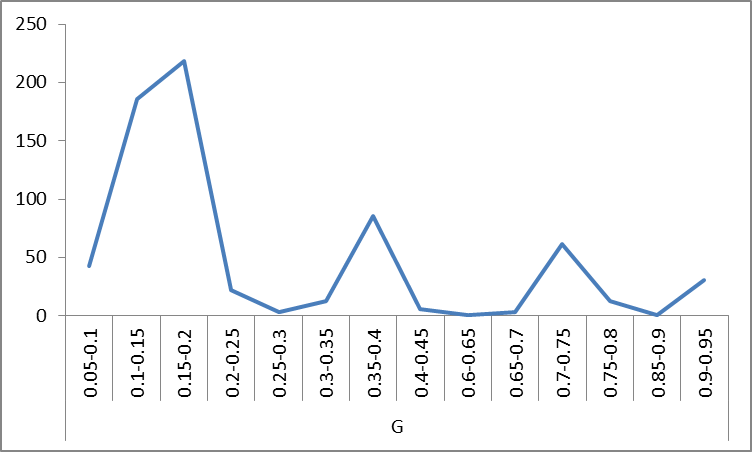


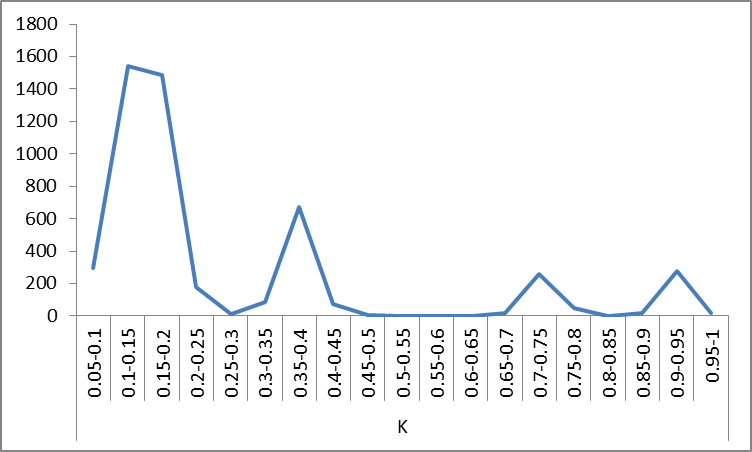


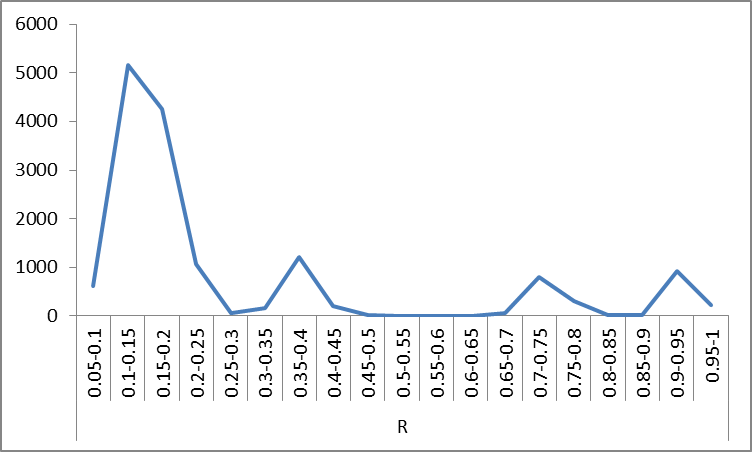


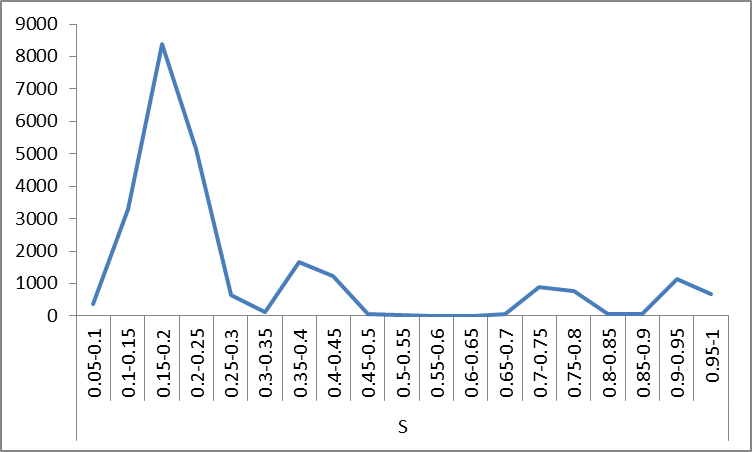


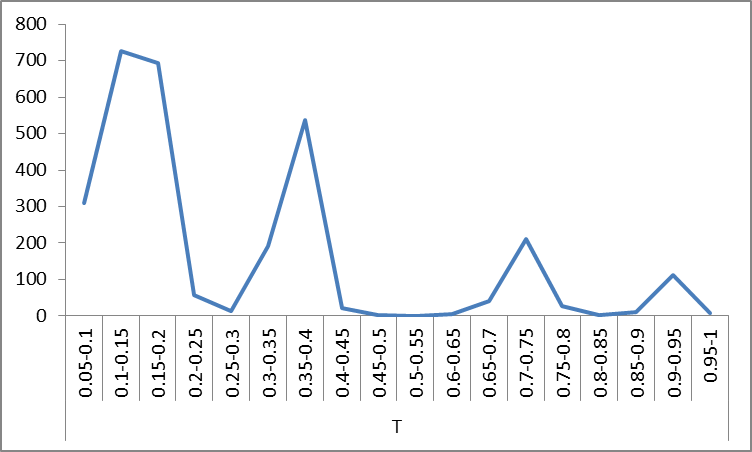


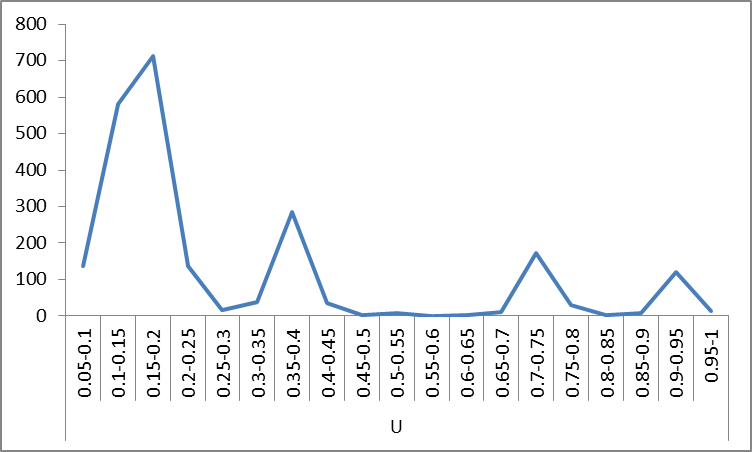


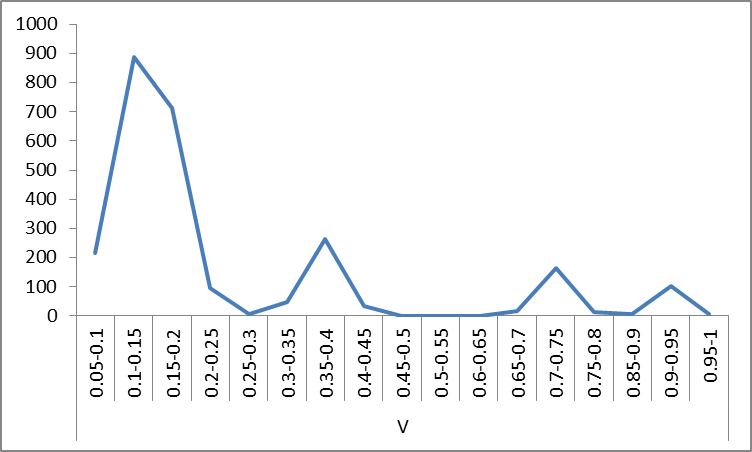


## C


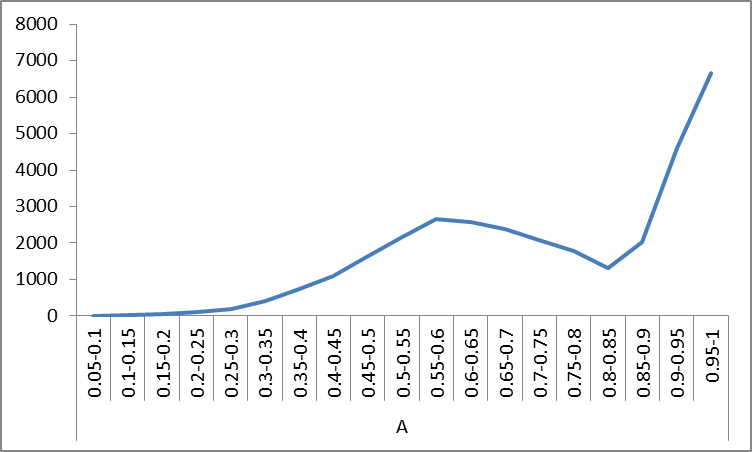


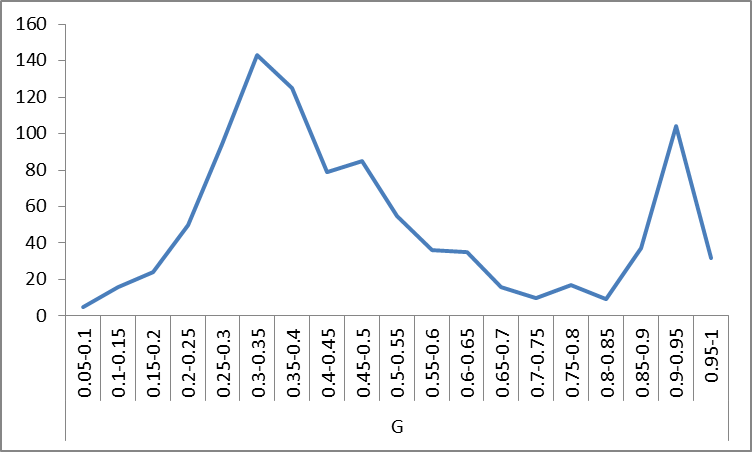


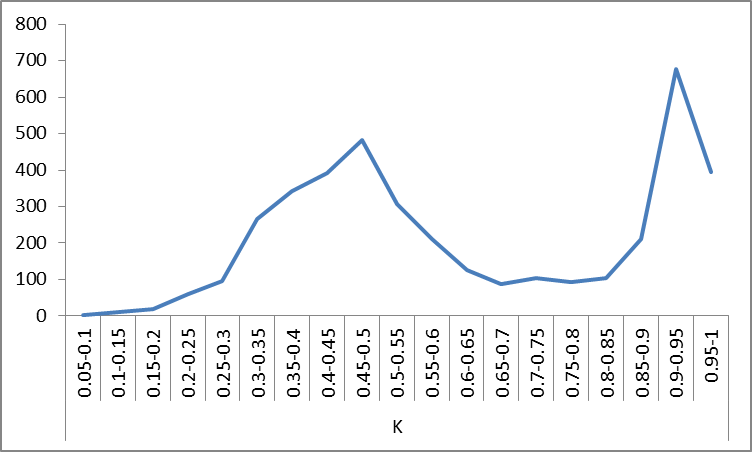


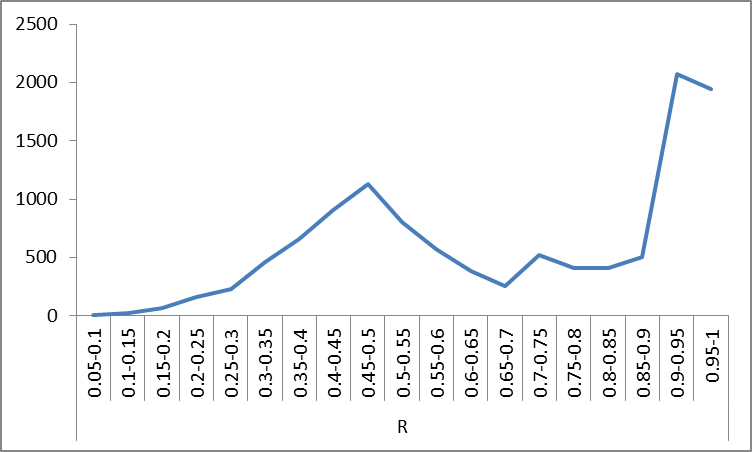


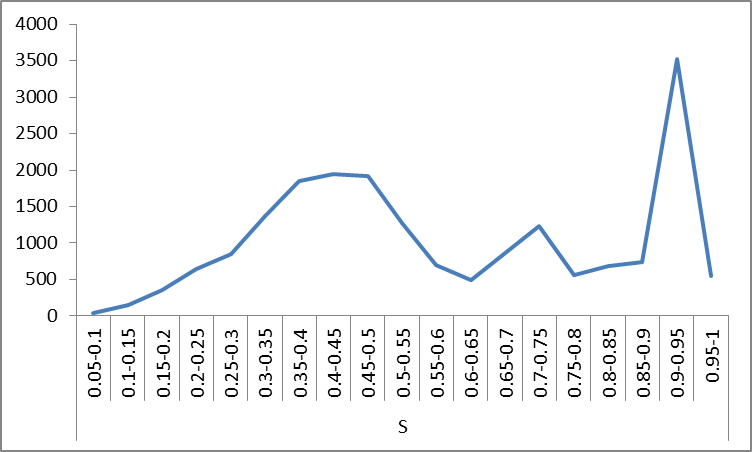


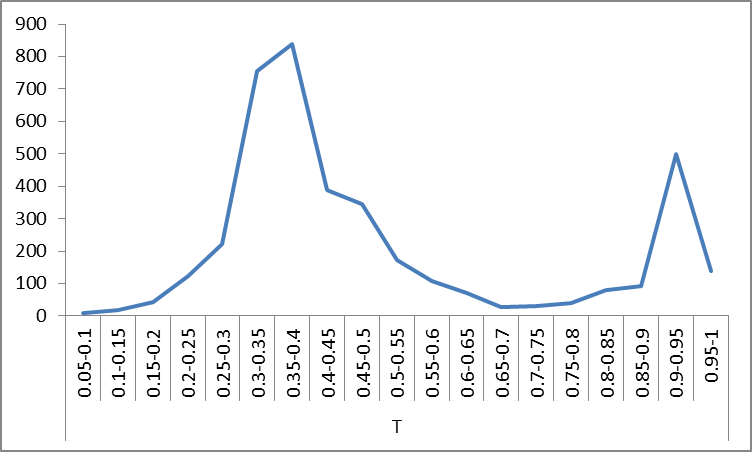


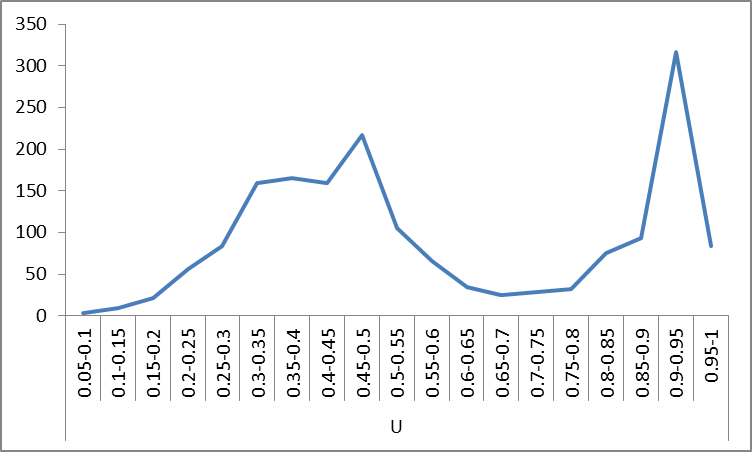


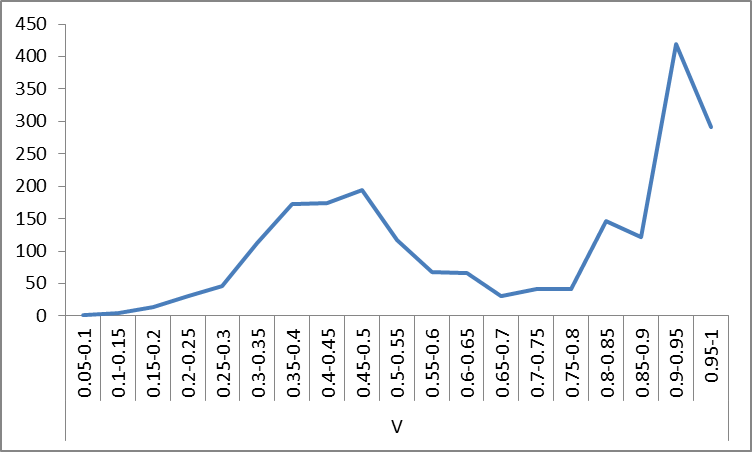

Supplement: Supplementary Materials S5 — The distributions of NMR CS data after normalization for shape strings. (DOC) [file pone.0083532.s005.doc]
