# Supplementary material for: NMRDSP: An Accurate Prediction of Protein Shape Strings from NMR Chemical Shifts and Sequence Data
Source: PLoS One. 2013 Dec 23;8(12):e83532. doi: 10.1371/journal.pone.0083532 (PMC3871590; doi:10.1371/journal.pone.0083532)
Supplement: Supplementary Materials S6 — The sequence identities in NS800 and NS203. (DOC) [file pone.0083532.s006.doc]

**Supplementary Materials**

**S6 The sequence identities distribution in NS800 and NS203**

**
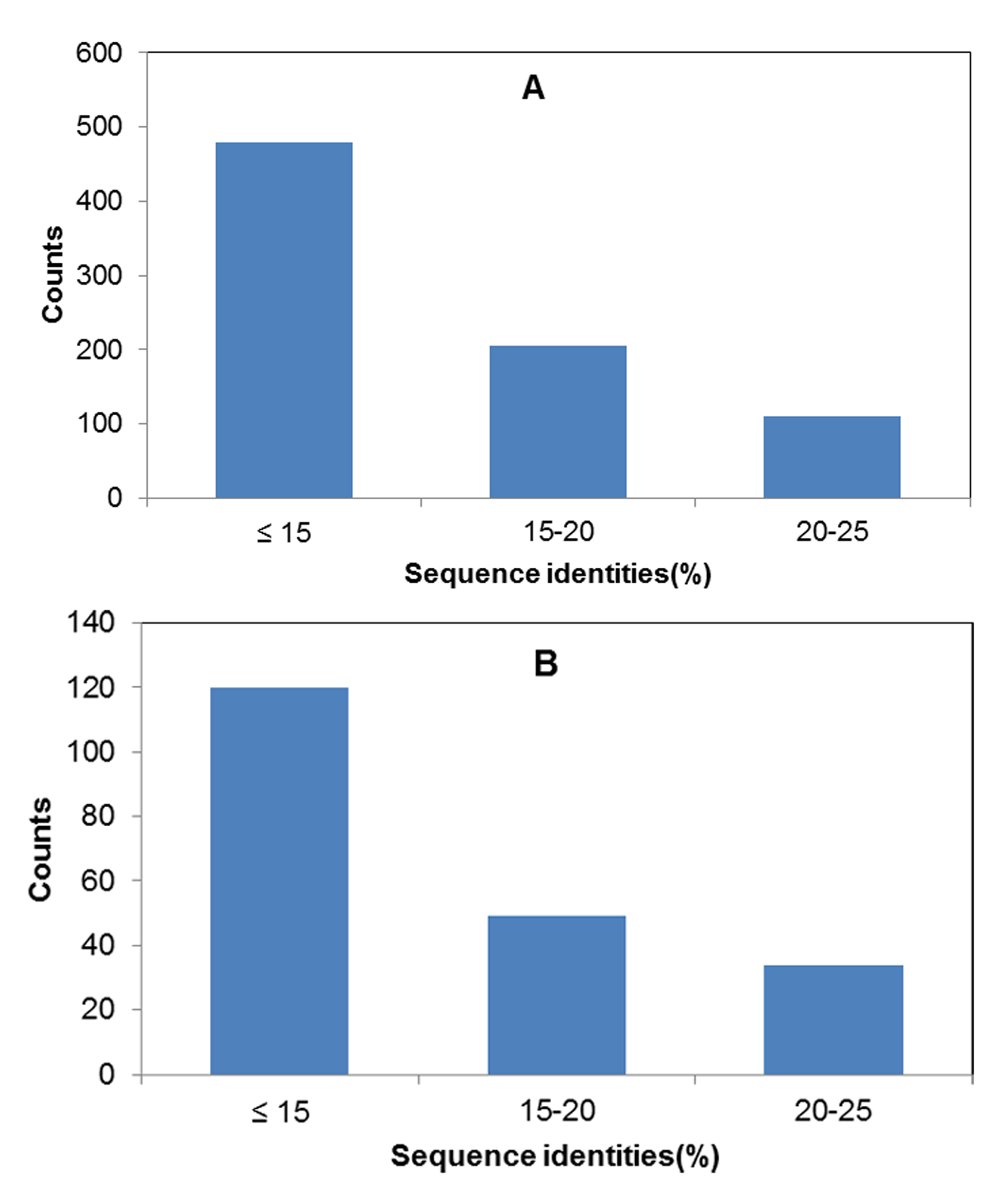
**

**Figure S6.1 (A) The distribution of the sequence identities in NS 800. (B) The distribution of the sequence identities in NS203.**

In Figure S6.1 (A), there are 480, 205 and 115 entries in the classes of ≤15%, 15%-20% and 20%-25% sequence identities of NS800. In Figure S6.1 (B), there are 120, 49 and 34 entries in the classes of ≤15%, 15%-20% and 20%-25% sequence identities of NS203.
