# Supplementary material for: NMRDSP: An Accurate Prediction of Protein Shape Strings from NMR Chemical Shifts and Sequence Data
Source: PLoS One. 2013 Dec 23;8(12):e83532. doi: 10.1371/journal.pone.0083532 (PMC3871590; doi:10.1371/journal.pone.0083532)
Supplement: Supplementary Materials S7 — The CRFs Template and SHIFTY format. (DOC) [file pone.0083532.s007.doc]

**Supplementary Materials**

**S7 The CRFs Template and SHIFTY format**

**Table S7.1 The CRFs Template**

| ·CRFs Template | | | |
| --- | --- | --- | --- |
| U0:%x[-3,0] | U1:%x[-2,0] | U2:%x[-1,0] | U3:%x[0,0] |
| U4:%x[1,0] | U5:%x[2,0] | U6:%x[3,0] | U7:%x[-3,1] |
| U8:%x[-2,1] | U9:%x[-1,1] | U10:%x[0,1] | U11:%x[1,1] |
| U12:%x[2,1] | U13:%x[3,1] | U14:%x[-3,2] | U15:%x[-2,2] |
| U16:%x[-1,2] | U17:%x[0,2] | U18:%x[1,2] | U19:%x[2,2] |
| U20:%x[3,2] | U21:%x[-3,3] | U22:%x[-2,3] | U23:%x[-1,3] |
| U24:%x[0,3] | U25:%x[1,3] | U26:%x[2,3] | U27:%x[3,3] |
| U28:%x[-3,4] | U29:%x[-2,4] | U30:%x[-1,4] | U31:%x[0,4] |
| U32:%x[1,4] | U33:%x[2,4] | U34:%x[3,4] | U35:%x[-3,5] |
| U36:%x[-2,5] | U37:%x[-1,5] | U38:%x[0,5] | U39:%x[1,5] |
| U40:%x[2,5] | U41:%x[3,5] | U42:%x[-3,6] | U43:%x[-2,6] |
| U44:%x[-1,6] | U45:%x[0,6] | U46:%x[1,6] | U47:%x[2,6] |
| U48:%x[3,6] | U49:%x[-3,7] | U50:%x[-2,7] | U51:%x[-1,7] |
| U52:%x[0,7] | U53:%x[1,7] | U54:%x[2,7] | U55:%x[3,7] |
| U56:%x[-3,8] | U57:%x[-2,8] | U58:%x[-1,8] | U59:%x[0,8] |
| U60:%x[1,8] | U61:%x[2,8] | U62:%x[3,8] | U63:%x[-3,9] |
| U64:%x[-2,9] | U65:%x[-1,9] | U66:%x[0,9] | U67:%x[1,9] |
| U68:%x[2,9] | U69:%x[3,9] | U70:%x[-3,10] | U71:%x[-2,10] |
| U72:%x[-1,10] | U73:%x[0,10] | U74:%x[1,10] | U75:%x[2,10] |
| U76:%x[3,10] | U77:%x[-3,11] | U78:%x[-2,11] | U79:%x[-1,11] |
| U80:%x[0,11] | U81:%x[1,11] | U82:%x[2,11] | U83:%x[3,11] |
| U84:%x[-3,12] | U85:%x[-2,12] | U86:%x[-1,12] | U87:%x[0,12] |
| U88:%x[1,12] | U89:%x[2,12] | U90:%x[3,12] | U91:%x[-3,13] |
| U92:%x[-2,13] | U93:%x[-1,13] | U94:%x[0,13] | U95:%x[1,13] |
| U96:%x[2,13] | U97:%x[3,13] | U98:%x[-3,14] | U99:%x[-2,14] |
| U100:%x[-1,14] | U101:%x[0,14] | U102:%x[1,14] | U103:%x[2,14] |
| U104:%x[3,14] | U105:%x[0,0] | U106:%x[0,1] | U107:%x[0,2] |
| U108:%x[0,3] | U109:%x[0,4] | U110:%x[0,5] | U111:%x[0,6] |
| U112:%x[0,7] | U113:%x[0,8] | U114:%x[0,9] | U115:%x[0,10] |
| U116:%x[0,11] | U117:%x[0,12] | U118:%x[0,13] | U119:%x[0,14] |

# SHIFTY Format


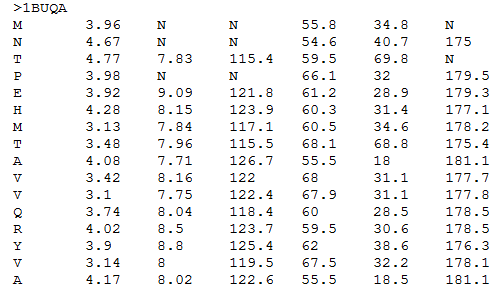


In the line just before your NMR data, protein PDB ID is necessary which is started with “>”. In the data, the first row is residue, the second row to the last one are chemical shifts of HA, H, N, CA, CB, C respectively. In the data, non-numeric strings (letters or other characters) are allowed to represent the unmeasured chemical shifts.
